# Supplementary material for: Using Dramatization to Teach Starling Forces in the Microcirculation to First-Year Medical Students
Source: MedEdPORTAL. 2019 Oct 18;15:10842. doi: 10.15766/mep_2374-8265.10842 (PMC6944257; doi:10.15766/mep_2374-8265.10842)
Supplement: Supplementary file 1 — A. Starling Forces Workshop Lecture.pptx B. Preactivity Quiz.docx C. Postactivity Quiz.docx D. Preactivity Quiz Answers.docx E. Postactivity Quiz Answers.docx [file mep-15-10842-s001.zip › _Educational_Summary_Report_10842.pdf]

MedEdPORTAL® The AAMC Journal of Teaching and Learning Resources

Original Publication Open Access

Using Dramatization to Teach Starling Forces in the Microcirculation to First-Year Medical Students

Brian William Connor, MD, Helena Carvalho, PhD*

*Corresponding author: helena@vt.edu

Abstract

Introduction: The clinical importance, prevalence, and multiple etiologies of tissue edema make it a critical part of medical education. Given the multiple physiological parameters that must be simultaneously considered to determine fluid movement, it is important that a deeper understanding of the microcirculation and fluid shifts is achieved in preclinical education. **Methods:** We describe an innovative teaching methodology using dramatization to interactively teach Starling forces to first-year medical students. Prior to the dramatization, students were given an introduction to Starling forces. They also completed a brief knowledge quiz on the topic before and after the activity. The classroom walls were marked with signs representing the intravascular space, extravascular or interstitium, and lymphatics compartments. Students were invited to act out or mimic the fluid shifts within capillaries as the values for hydrostatic and colloid osmotic pressures for the intravascular and interstitial spaces were presented. The goal was for each student to decide which compartment he/she would move to as fluid according to Starling force values and/or clinical scenarios. **Results:** A significant improvement between pre- and postactivity quiz performance (45.4% ± 25.1% and 77.5% ± 14.1%, respectively) was observed ($n = 26$, $p < .001$, *t* test). In a postactivity survey, 85% of students reported the activity to be an effective way of learning. **Discussion:** Our data indicate that this dramatization approach is effective in complementing passive learning in traditional lectures. Furthermore, this type of dynamic activity brings joy to the classroom and breaks the monotony of lecturing.

Keywords
Microcirculation, Active Learning, Edema, Fluid Movement, Hydrostatic Pressure, Colloid Osmotic Pressure

Educational Objectives

By the end of this classroom dramatization activity, learners will be able to:

1. Define how Starling forces affect both filtration and reabsorption at the level of the microcirculation.
2. Participate in an interactive exercise to learn the key players involved in fluid movement within the microcirculation.
3. Apply the concept of Starling forces to clinically relevant scenarios and describe how this concept relates to the multiple etiologies of tissue edema.

Citation:
Connor BW, Carvalho H. Using dramatization to teach Starling forces in the microcirculation to first-year medical students. *MedEdPORTAL*. 2019;15:10842. https://doi.org/10.15766/mep_2374-8265.10842

Introduction

Edema is a common clinical manifestation of disease; therefore, medical students should have a deep understanding of how physiological and pathological changes in vascular permeability and colloid and osmotic pressures affect filtration and absorption. A fuller understanding of these factors and their roles in fluid distribution can help the student appreciate the multiple etiologies of tissue edema to determine the best course of management. The concept of Starling forces, which determine fluid distribution, is often taught to first-year medical students within traditional didactic lectures.

Lecture is a great tool for knowledge transmission of large amounts of content from faculty to students. The challenge is for students to be able to assimilate this ever-growing knowledge; consequently, many elect to memorize instead of understand. There are various ways to adapt a lecture,[1] making it memorable as it engages students. We recognize the need to develop and implement alternative teaching methodologies to provide support for all types of learners. In an attempt to promote

Copyright © 2019 Connor and Carvalho. This is an open-access publication distributed under the terms of the Creative Commons Attribution license.

long-term learning, we describe an active classroom exercise that can be used to complement or replace a traditional lecture. After seeing the positive impact of using dramatization to teach other cardiovascular concepts,[2,3] we developed and implemented a dramatization to teach Starling forces and their application to clinical scenarios.

Acknowledging that lecture attendance has been declining in favor of other resources, we wanted to deliver an engaging activity that would promote learning through visual, auditory, and kinesthetic modalities; the last was particularly pertinent given that it involved physical movement—one essential aspect of dramatization. The activity could lead to peer-to-peer interaction, promote teamwork, and stimulate humor that could contribute to learning.[4] In addition, the activity did not require specialized equipment or space, making it inexpensive, deliverable in most types of classrooms, and easily completed in a relatively short amount of time to facilitate flexibility in terms of curricular implementation.

Although simulation exercises involving clinical encounters are currently published in *MedEdPORTAL*,[5] including some simulations that address edema[6] and the microcirculation,[7] to the best of our knowledge, there are no publications in *MedEdPORTAL* using dramatization in the manner described here. This report not only presents an innovative teaching methodology (dramatization) but also addresses the basic science concepts underlying the important physiological mechanism of Starling forces in the microcirculation that should be understood fully by first-year medical students.

Methods

Virginia Tech Carilion School of Medicine (VTCSOM) has a hybrid problem-based learning (PBL) curricular model. For the first 2 years of the curriculum (preclerkship), the content is offered in eight blocks: Blocks I-IV (8 weeks each) during the first year and Blocks V-VIII (6 weeks each) in the second year. Student learning in basic science is facilitated through a variety of activities, such as PBL, hands-on workshops, and lectures. Our dramatization activity was delivered during Block II, which covers cardiovascular, respiratory, and skeletal muscle. No major prerequisite knowledge or prior active learning experience was necessary for students to participate in the session. Additionally, student prereading was encouraged but not required. Instructors had to be familiar with vascular organization, vascular permeability, hydrostatic pressure, and colloid oncotic pressure.

Prior to the activity, we designated areas of the classroom to represent the following compartments: intravascular, extravascular or interstitial spaces, and lymphatics. Although it could be done in different ways, we simply fixed paper signs to each area/compartment.

At the beginning of a 50-minute session, we gave a short presentation (5-10 minutes; Appendix A) to review the microcirculation compartments (arteriolar, capillary, venular, interstitial space) and the physical principles of fluid movement and introduce Starling forces and the Starling equation. Following the basic review of concepts, we asked students to mimic the movement of fluid in response to the different scenarios. The students had the choice to actively participate or simply observe their peers, and this was agreed on prior to starting the activity to avoid frustration or misunderstandings. Ultimately, all 26 students chose to actively participate. The activity started with the projection of the simple values for the four forces: (1) intravascular hydrostatic, (2) intravascular colloid osmotic, (3) extravascular hydrostatic, and (4) extravascular colloid osmotic. Three students started from a designated space named *intravascular*, and three additional students started from a designated space named *interstitium.* Students were instructed to stay in or move out of their respective areas to demonstrate where they thought fluid would move after evaluating the four Starling force values provided, switching student volunteers between each of the scenarios presented to allow all students to participate. Once students understood the dynamics of the exercise, we presented them with more challenging tasks, using vignettes to make the activity clinically relevant. At this point, the students had to compete to move faster than their peers from the circulation (intravascular) to the interstitium or vice versa as a response to patient data projected using a variety of scenarios (Appendix A). For example, as the plasma protein concentration declined (e.g., due to hepatic failure or renal loss of protein), students had to deduce that the colloid osmotic pressure would decline, leading to more filtration and loss of fluid from the plasma to the interstitium. The correct response, in this scenario, was to move out (or filter out) of the intravascular compartment to the interstitial compartment. The exercise continued as the scenarios varied, and the students continued to act as fluid and physically moved to mimic reabsorption into the capillary or filtration into the interstitium or excess fluid removal by the lymphatics.

We used student performance on pre- and postactivity quizzes (Appendices B and C; answer keys in Appendices D and E, respectively) as a measure of the activity's effectiveness. It

Copyright © 2019 Connor and Carvalho. This is an open-access publication distributed under the terms of the Creative Commons Attribution license.

was emphasized that the quiz was anonymous and voluntary, would not affect the students' grade, and was approved by the Virginia Tech institutional review board. The quizzes were a combination of 11 short-answer, multiple-choice, and true/false questions regarding basic physiology, pathology, and higher-level application of the effects of Starling forces on the microcirculation. We developed these quiz questions (pilot tested by five second-year medical students) based on concepts emphasized in the VTCSOM end-of-block examination. We also had one question on the quiz (question 8 prequiz, question 9 postquiz) not covered nor explained by learning the material in the activity (which would flag us of other possible issues). In addition, the question order was randomized between pre- and postactivity surveys to avoid simple repetition of the same answer in the same place. Students placed the survey, complete or not, in an envelope that circulated through the classroom, providing anonymity. To pair the results, the pre- and postactivity scores were matched by asking students to mark each quiz with an individualized code. We emphasized that students should leave a question blank if they were unsure of the answer because if they were to correctly guess the response, it would impact our evaluation of overall activity effectiveness.

Results

The implementation of the activity was well received. Students had the option to only observe or participate in the dramatization, and all 26 students in attendance participated and responded to the pre- and postquizzes. In the postsurvey, 85% of students found this activity to be an effective way of learning. Fifteen open-ended comments were obtained from the 26 student participants. There were no negative comments. Comments provided centered around themes suggesting that the dramatization made the complex topic of Starling forces easier to grasp; technical recommendations for future dramatizations; the helpful nature of the vignettes provided; and the simplicity that can be imparted by acting out concepts in the future, regardless of their innate complexity.

Students' performance on the postactivity quiz (77.5% ± 14.1%) was significantly higher ($p < .001$, t test) than the preactivity quiz performance (45.4% ± 25.1%; Figure 1). Performance on all items except prequiz question 8 (the internal control that was a topic unrelated to the teaching) showed improved group performance after the activity (Figure 2). The lack of improvement on prequiz question 8 (postquiz question 9), which was not addressed during the activity, confirmed that the improvement on all other quiz items reflected student learning and not merely luck, possible cheating, and so on. In addition, the range of responses (reflecting variation in students' understanding) was broader before (range: 9%-91%) as compared to after (range: 45%-100%) the activity.

Of note, not all questions revealed a large improvement between pre- and postactivity quizzes. Prequiz question 6 (postquiz question 10) was a true/false question in which students were

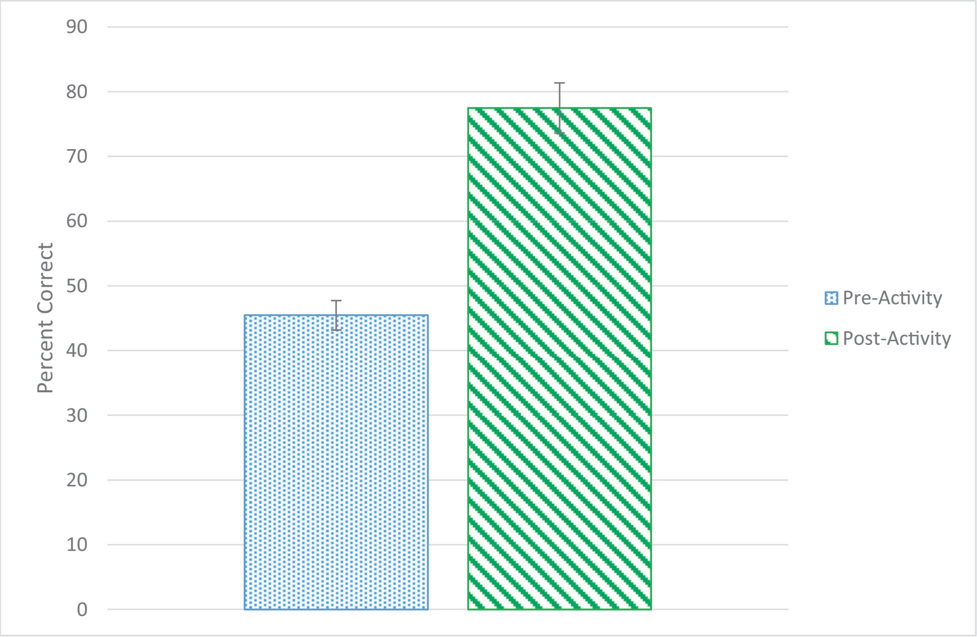

Figure 1. Overall student performance on the pre- and posttest as percentage of correct responses. Error bars represent the standard error of the mean.

Copyright © 2019 Connor and Carvalho. This is an open-access publication distributed under the terms of the Creative Commons Attribution license.

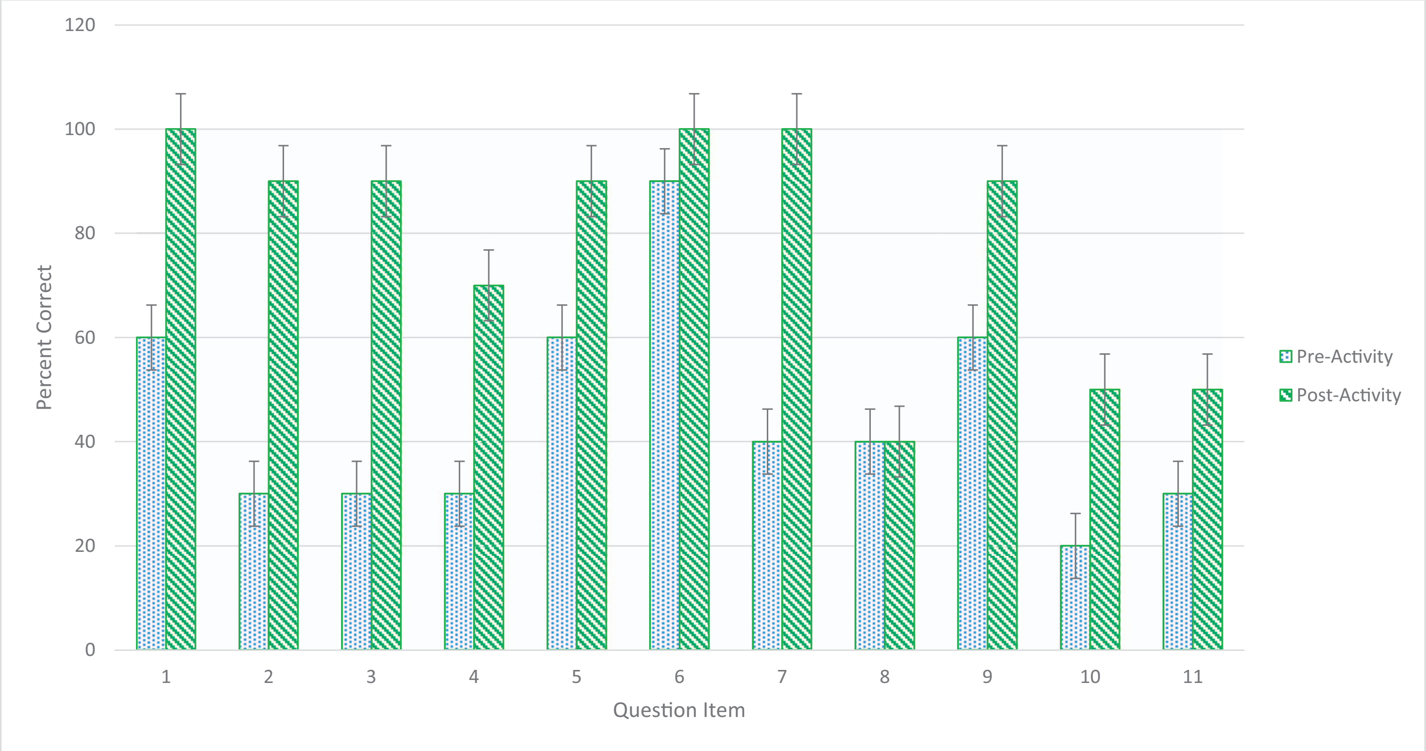

Figure 2. Student performance by question: responses to individual questions on the prequiz before the dramatization and the postquiz at the end of the class activity as percentage of correct responses. Error bars represent the standard error of the mean.

asked about the mechanism by which the lymphatics absorb excess fluid from surrounding tissues. Although covered in the activity, the mechanism of fluid removal by the lymphatics was perhaps less emphasized. In addition, prequiz questions 10 and 11 (postquiz questions 6 and 5, respectively) comparatively had somewhat overall low correct response rates. These items were application questions that, although not directly covered in the workshop, could have been solved by higher-level application of a basic knowledge fund imparted by the dramatization.

Discussion

We presented an alternative teaching style for the first-year medical student that is an effective method of teaching Starling forces within the microcirculation and this concept's implication in the development of edema. We noted a significant improvement in knowledge when comparing the results of the postactivity quiz with those of the preactivity quiz, suggesting the overall efficacy of this learning approach. We received positive comments from the participants, who stated that the activity helped them identify missing concepts while also making the teaching more interesting. This approach can be used in substitution for traditional didactics or to complement a lecture, as in our case.

Another valuable aspect is accessibility: This activity is virtually free of costs, as it does not require any special equipment. It also can be offered within different sizes of classroom, as it is flexible in terms of space and the number of student participants. For a very large classroom, a group of volunteer students would participate, and the other students would observe and learn by watching their peers, potentially alternating roles.

Our previous experience with traditional lecture revealed that some students become confused with the four different physiological forces that apply to the Starling equation. As a consequence, some students tend to elect to memorize the formula. As a result, those learners may be able to resolve a question but will ultimately lack a deeper understanding of this common and clinically relevant phenomenon.

This activity was originally developed by an M1 student at the time (Brian William Connor) for his small group in PBL, inspired by another dramatization[2,3] offered by a faculty member in the classroom (Helena Carvalho; cardiac cycle dramatization). The excitement and engagement observed in the PBL group prompted further development to be offered to all students. Considering the importance of understanding edema

Copyright © 2019 Connor and Carvalho. This is an open-access publication distributed under the terms of the Creative Commons Attribution license.

formation, its complications, and the various factors involved in its regulation, we decided to offer this innovative teaching strategy to the entire class, where students would be involved and actively engaged, with the hope of making the learning process memorable, which we achieved as supported by our subjective student feedback.

Some observations from the activity should be taken into consideration. For example, most students felt very comfortable moving to act as fluid in front their peers, whereas others did not. In our opinion, it is important for all students to learn without the pressure that some might feel when in front of a classroom of peers. For the most extroverted students, this activity is an opportunity to enjoy a game and interact in a dynamic way in the classroom setting, whereas shy students have the pleasure of watching peers interact without any type of embarrassment imposed on them. In our cohort, all students volunteered, even though some were more expressive than others. Facilitators should not be shy, as the activity can be viewed as a game where enjoyment is an expected outcome.

Limitations were mainly due to the small sample size. Eventually, we would like to be able to make well-supported inferences as to which part of the content was better served by this activity as well as to evaluate the activity's effect on long-term learning. However, we did observe an overall improvement in knowledge, with improvement in correct responses on each item on the postactivity quiz as compared to preactivity performance, with the expected exception of our control question (not covered in our dramatization), on which there was no difference between pre- and postactivity performance. Ideally, this dramatization would be tested with a larger group to better evaluate its overall effectiveness. A major challenge to confirming how well it works is the lack of a pure control (the same cohort of students' preactivity knowledge served as the control). Furthermore, the effectiveness of this activity in regard to long-term learning and concept solidification was not assessed in our study. In our medical school, there is only one first-year class, and keeping the best interests of the learner in mind, we did not divide the class in half to teach a traditional lecture to one cohort and the dramatization to the second, as would be necessary to compare their performances after each respective session and long-term retention of the concepts.

Future directions for this work include repetition of this kinetic learning workshop for first-year medical students, thereby increasing the sample size and allowing for continued evaluation of the workshop's demonstrated efficacy. Additionally, incorporating this or a similar workshop in the second-year medical school curriculum would provide an insight as to how performance changes on higher-level and even pathology-based questions (i.e., prequiz questions 10 and 11) relating to the effect of Starling forces on the microvasculature. Based on our open-ended feedback, it seems that current medical students appreciate various teaching methodologies—of which kinetic learning is just one—to break up the monotony of what can amount to hours of traditional didactic lectures. Thus, through this example, we hope to inspire medical educators to adopt less traditional approaches to teaching to enhance student participation and, ultimately, knowledge retention. If educators use this type of learning approach with their own students (to teach Starling forces or other physiological concepts), we encourage them to share with us their experiences with this alternative learning modality.

Appendices

A. Starling Forces Workshop Lecture.pptx
B. Preactivity Quiz.docx
C. Postactivity Quiz.docx
D. Preactivity Quiz Answers.docx
E. Postactivity Quiz Answers.docx

All appendices are peer reviewed as integral parts of the Original Publication.

Brian William Connor, MD: Medical Student, Virginia Tech Carilion School of Medicine

Helena Carvalho, PhD: Associate Professor, Department of Basic Science Education, Virginia Tech Carilion School of Medicine

Disclosures
None to report.

Funding/Support
None to report.

Prior Presentations
Connor BW, Carvalho H. Dramatization of Starling forces: an interactive learning approach. Paper presented at: International Association of Medical Science Educators Annual Meeting; June 8, 2016; Leiden, The Netherlands.

Ethical Approval
Reported as not applicable.

Copyright © 2019 Connor and Carvalho. This is an open-access publication distributed under the terms of the Creative Commons Attribution license.

References

1. Richardson D. Don't dump the didactic lecture; fix it. *Adv Physiol Educ*. 2008;32(1):23-24. https://doi.org/10.1152/advan.00048.2007
2. Carvalho H. A group dynamic activity for learning the cardiac cycle and action potential. *Adv Physiol Educ*. 2011;35(3):312-313. https://doi.org/10.1152/advan.00128.2010
3. Dowlati E, Musick DW, Zhang L, Thornton K, Carvalho H. Use of dramatization to teach cardiac cycle physiology to medical students. *J Educ Train Stud*. 2016;4(9):100-108. https://doi.org/10.11114/jets.v4i9.1603
4. Lujan HL, DiCarlo SE. Humor promotes learning! *Adv Physiol Educ.* 2016;40(4):433-434. https://doi.org/10.1152/advan.00123.2016
5. Keenan MP, Schenker KA, Sarsfield MJ. A complicated opioid overdose: a simulation for emergency medicine residents. *MedEdPORTAL*. 2017;13:10616. https://doi.org/10.15766/mep_2374-8265.10616
6. Paolo W. High-fidelity simulation case: teaching diabetic ketoacidosis with cerebral edema. *MedEdPORTAL*. 2014;10:9769. https://doi.org/10.15766/mep_2374-8265.9769
7. Ressetar H, Allen E. Histology of the liver lobule. *MedEdPORTAL*. 2010;6:7990. https://doi.org/10.15766/mep_2374-8265.7990

Received: February 4, 2019
Accepted: July 9, 2019
Published: October 18, 2019

Copyright © 2019 Connor and Carvalho. This is an open-access publication distributed under the terms of the Creative Commons Attribution license.
