# Supplementary material for: Using Dramatization to Teach Starling Forces in the Microcirculation to First-Year Medical Students
Source: MedEdPORTAL. 2019 Oct 18;15:10842. doi: 10.15766/mep_2374-8265.10842 (PMC6944257; doi:10.15766/mep_2374-8265.10842)
Supplement: Supplementary file 1 — A. Starling Forces Workshop Lecture.pptx B. Preactivity Quiz.docx C. Postactivity Quiz.docx D. Preactivity Quiz Answers.docx E. Postactivity Quiz Answers.docx [file mep-15-10842-s001.zip › A. Starling Forces Workshop Lecture.pptx]

## Slide 1
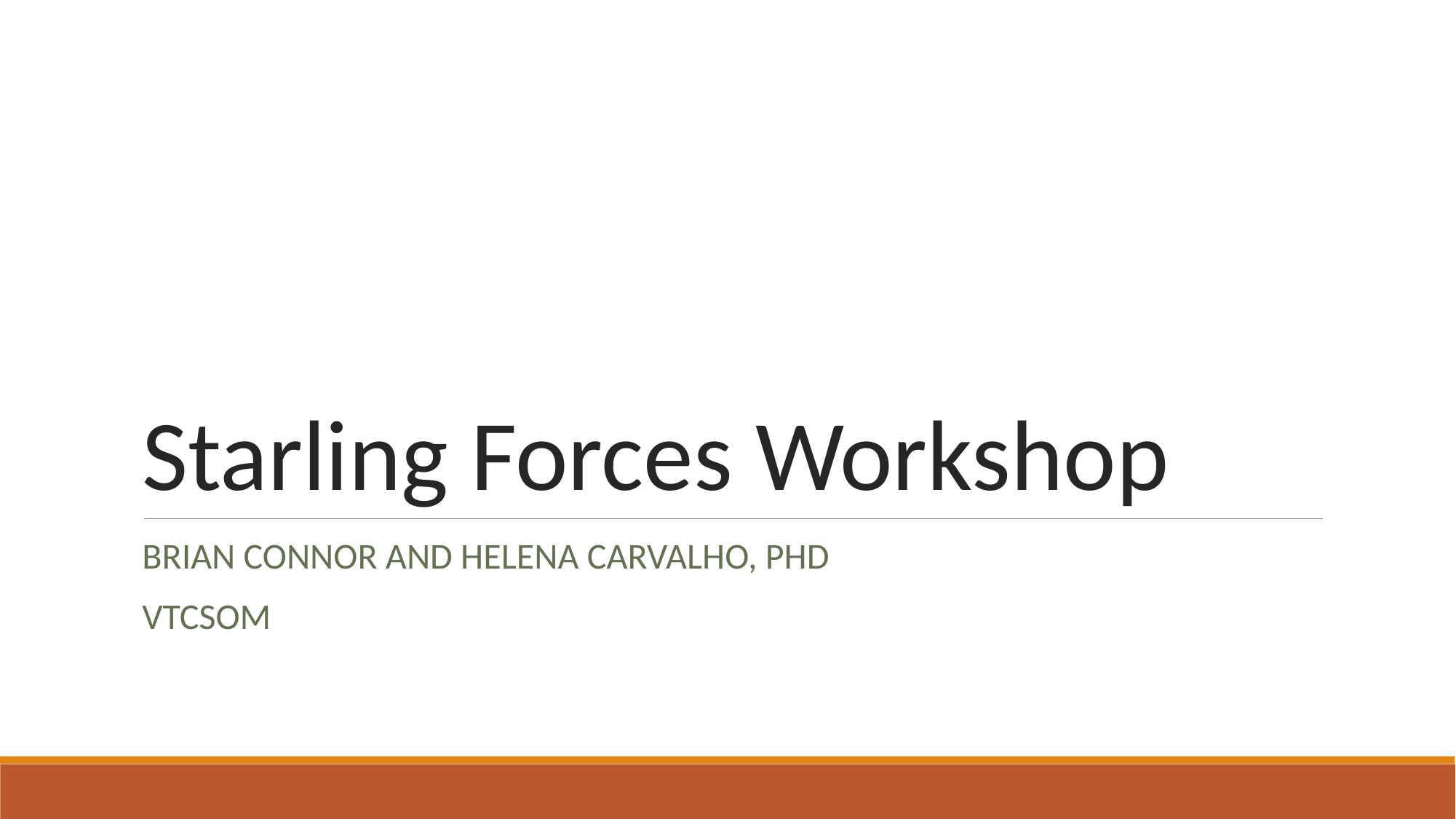

# Starling Forces Workshop
BRIAN CONNOR AND HELENA CARVALHO, PHD
VTCSOM

## Slide 2
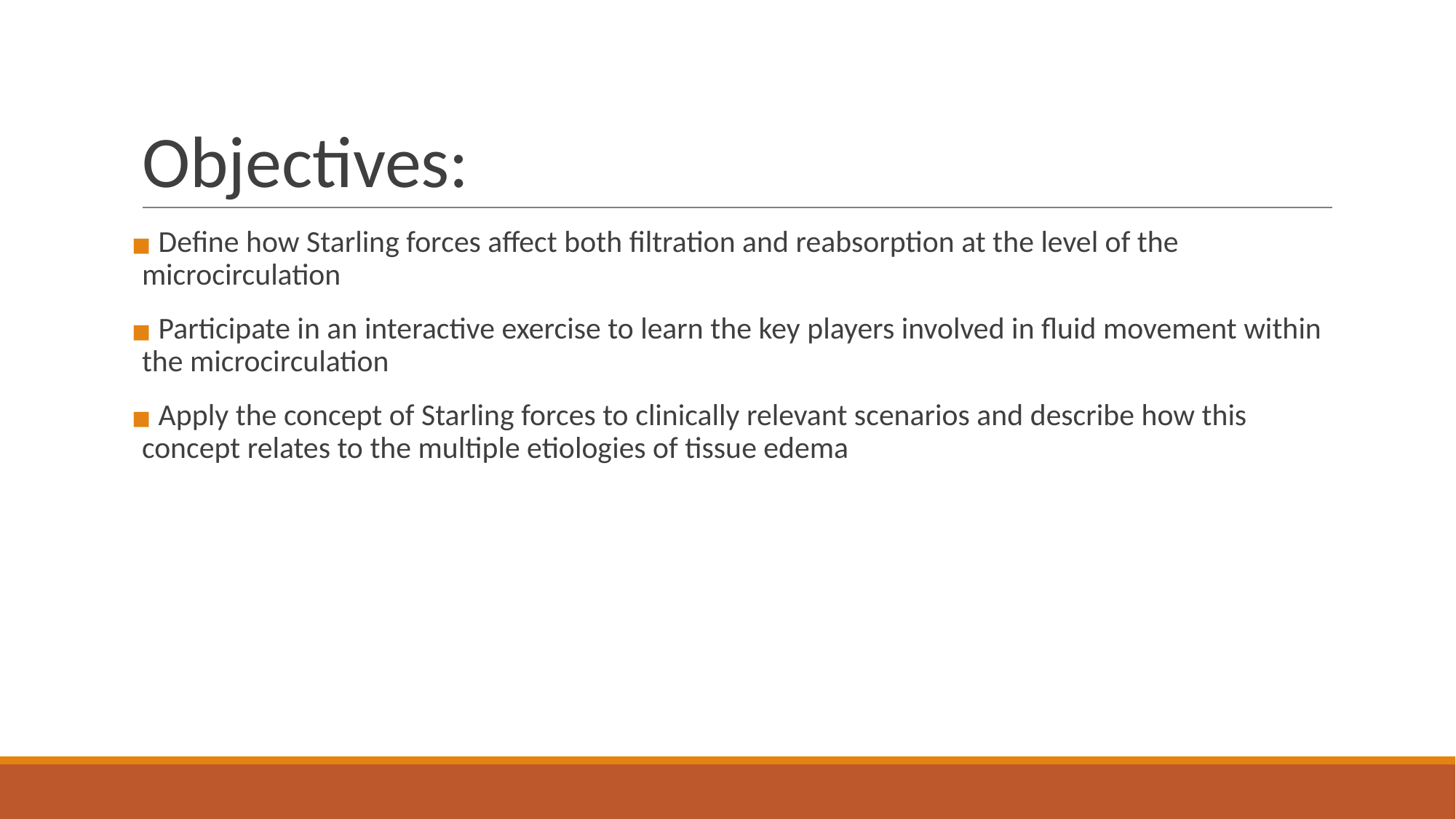

# Objectives:
 Define how Starling forces affect both filtration and reabsorption at the level of the microcirculation
 Participate in an interactive exercise to learn the key players involved in fluid movement within the microcirculation
 Apply the concept of Starling forces to clinically relevant scenarios and describe how this concept relates to the multiple etiologies of tissue edema

## Slide 3
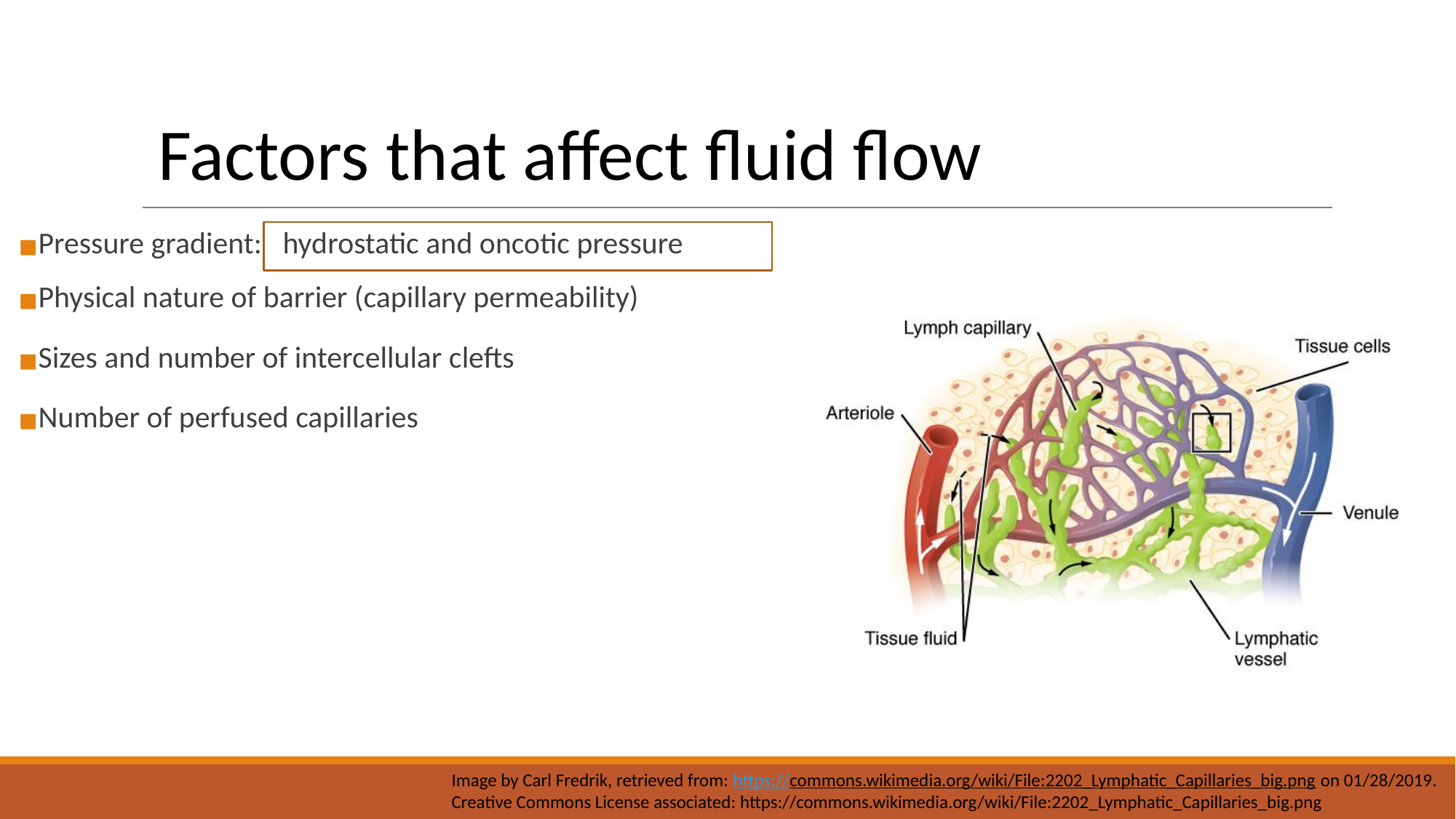

# Factors that affect fluid flow
Pressure gradient: hydrostatic and oncotic pressure
Physical nature of barrier (capillary permeability)
Sizes and number of intercellular clefts
Number of perfused capillaries
Image by Carl Fredrik, retrieved from: https://commons.wikimedia.org/wiki/File:2202_Lymphatic_Capillaries_big.png on 01/28/2019. Creative Commons License associated: https://commons.wikimedia.org/wiki/File:2202_Lymphatic_Capillaries_big.png

## Slide 4
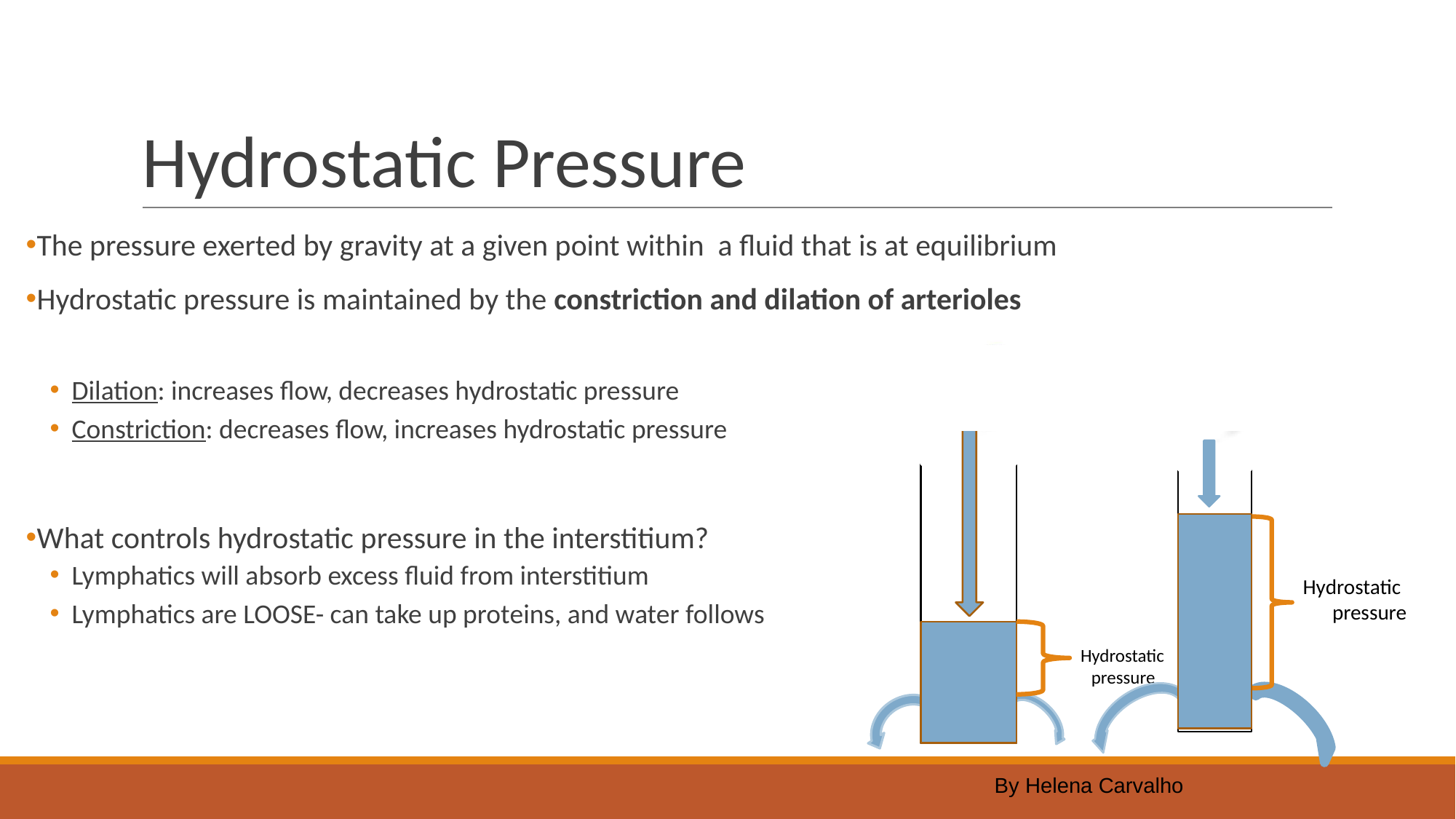

# Hydrostatic Pressure
The pressure exerted by gravity at a given point within a fluid that is at equilibrium
Hydrostatic pressure is maintained by the constriction and dilation of arterioles
Dilation: increases flow, decreases hydrostatic pressure
Constriction: decreases flow, increases hydrostatic pressure
What controls hydrostatic pressure in the interstitium?
Lymphatics will absorb excess fluid from interstitium
Lymphatics are LOOSE- can take up proteins, and water follows
Hydrostatic
pressure
Hydrostatic
pressure
By Helena Carvalho

## Slide 5
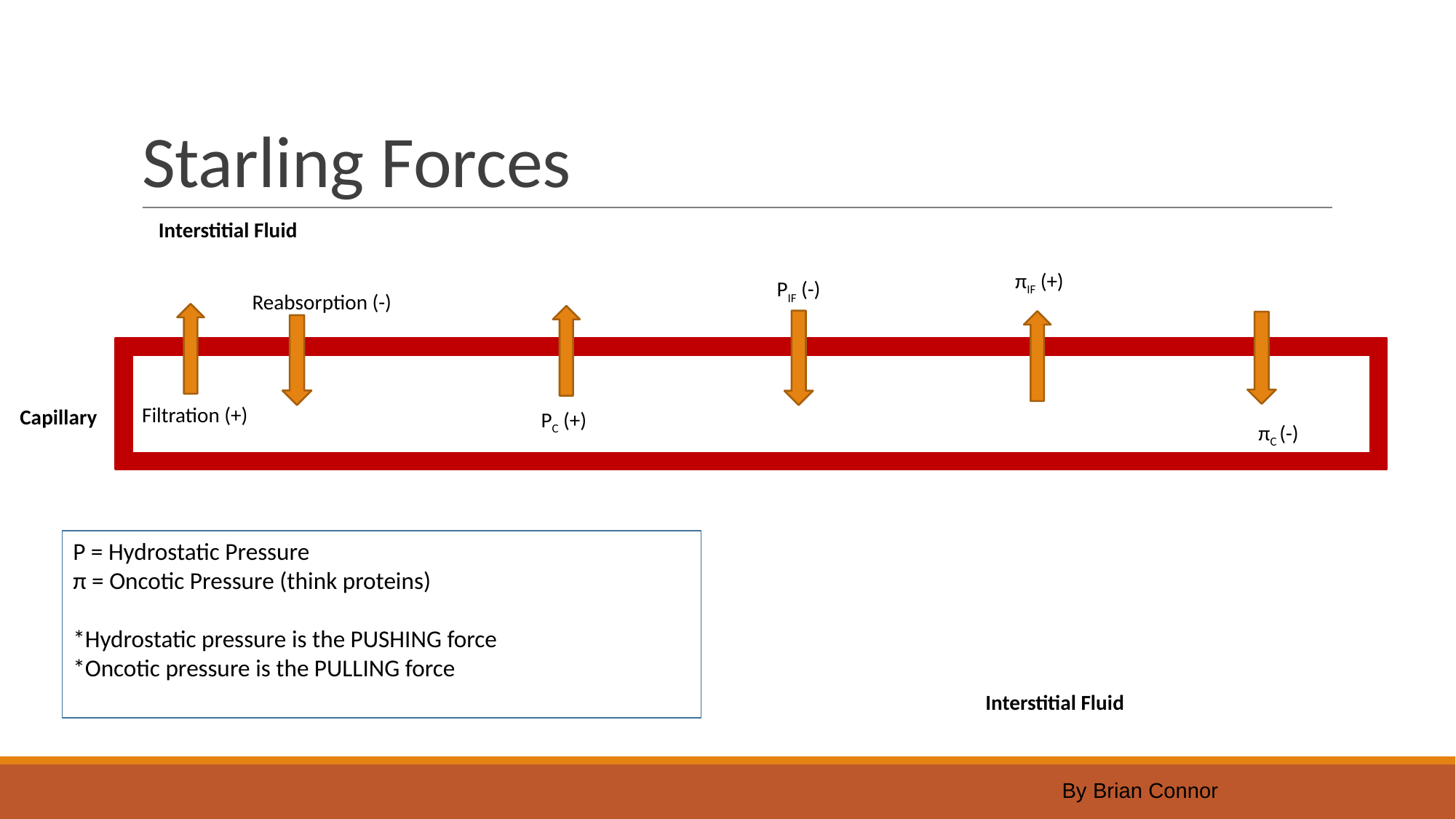

# Starling Forces
Interstitial Fluid
πIF (+)
PIF (-)
Reabsorption (-)
Filtration (+)
Capillary
PC (+)
πC (-)
P = Hydrostatic Pressure
π = Oncotic Pressure (think proteins)
*Hydrostatic pressure is the PUSHING force
*Oncotic pressure is the PULLING force
Interstitial Fluid
By Brian Connor

## Slide 6
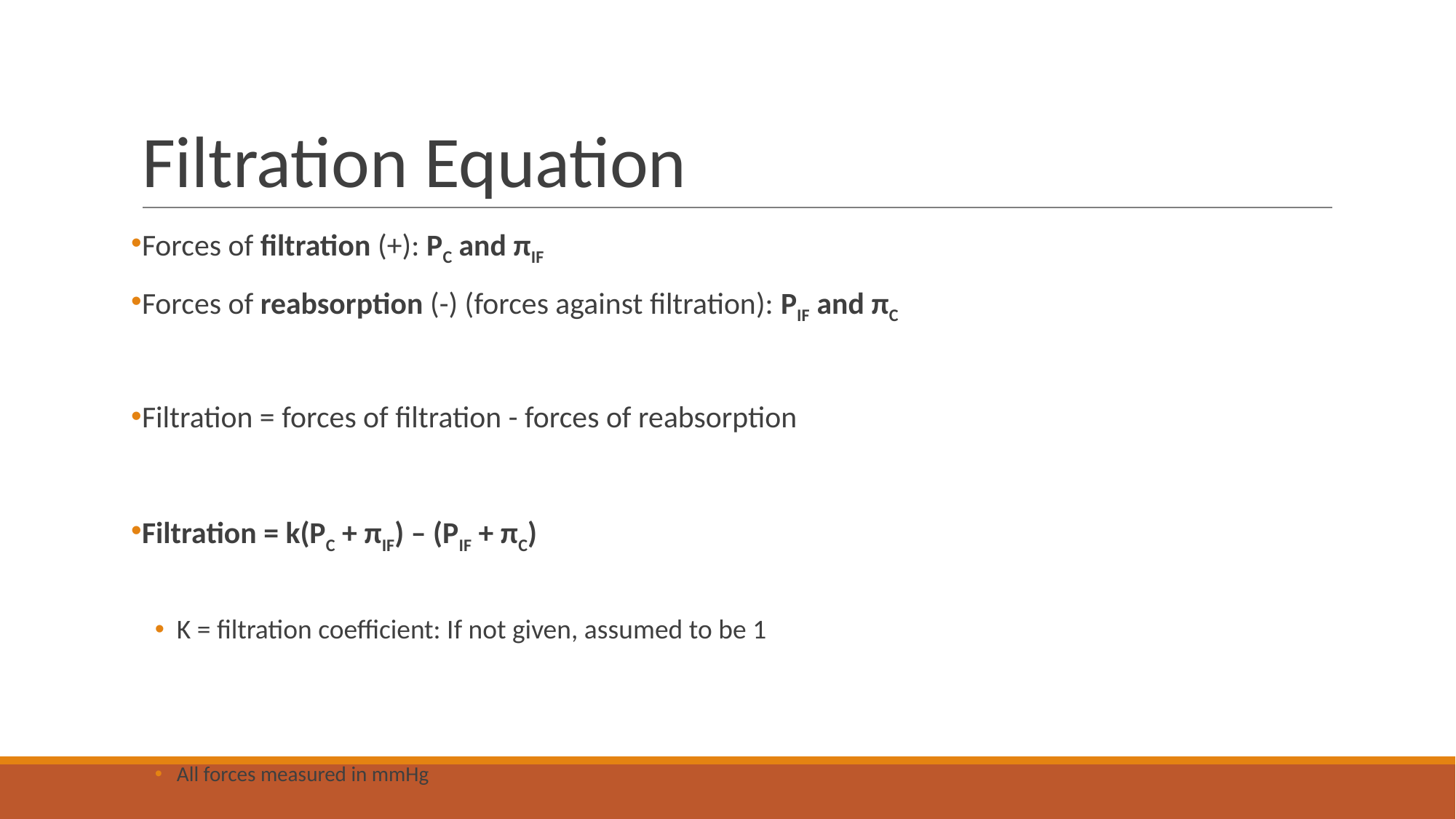

# Filtration Equation
Forces of filtration (+): PC and πIF
Forces of reabsorption (-) (forces against filtration): PIF and πC
Filtration = forces of filtration - forces of reabsorption
Filtration = k(PC + πIF) – (PIF + πC)
K = filtration coefficient: If not given, assumed to be 1
All forces measured in mmHg

## Slide 7
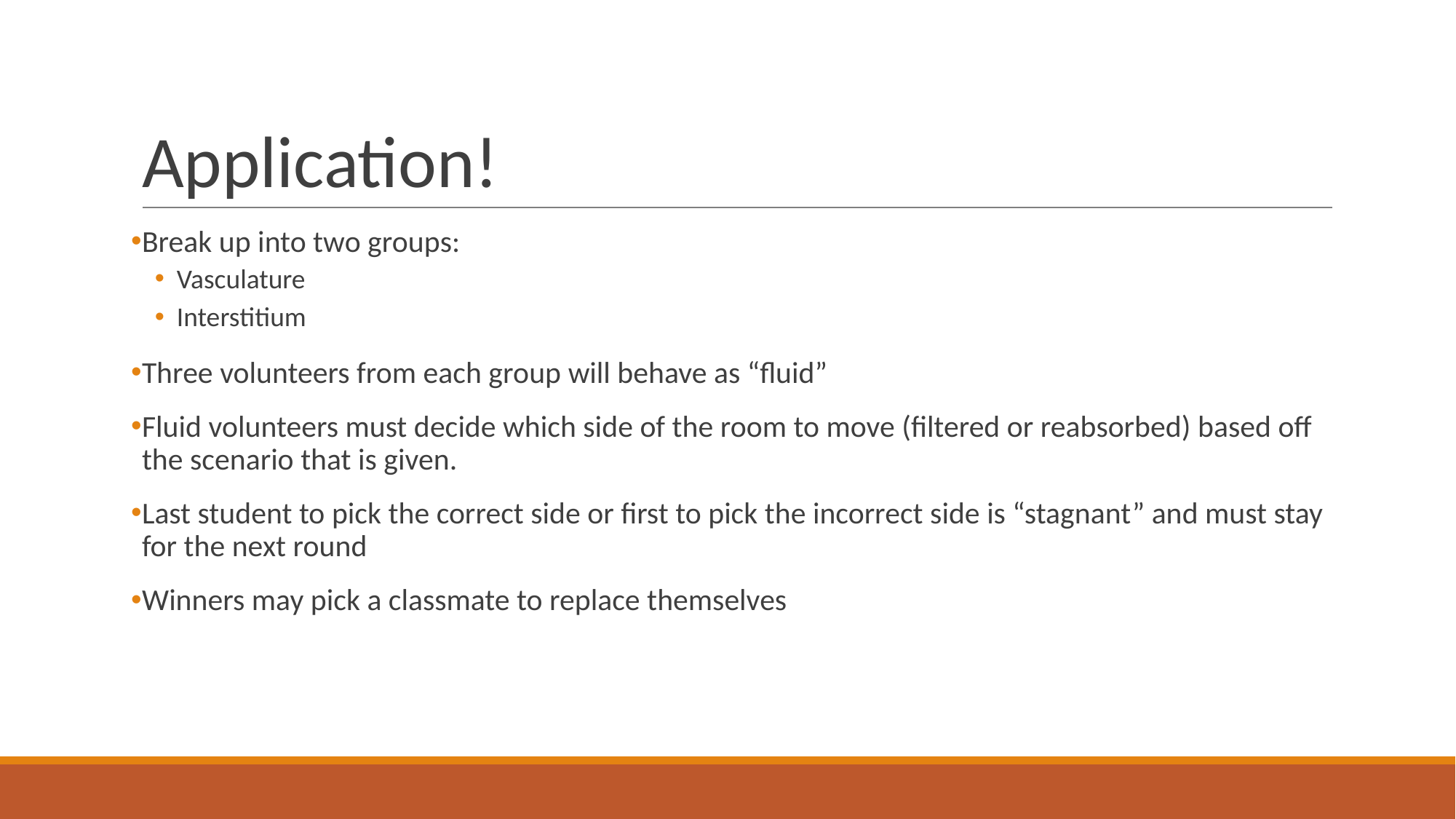

# Application!
Break up into two groups:
Vasculature
Interstitium
Three volunteers from each group will behave as “fluid”
Fluid volunteers must decide which side of the room to move (filtered or reabsorbed) based off the scenario that is given.
Last student to pick the correct side or first to pick the incorrect side is “stagnant” and must stay for the next round
Winners may pick a classmate to replace themselves

## Slide 8
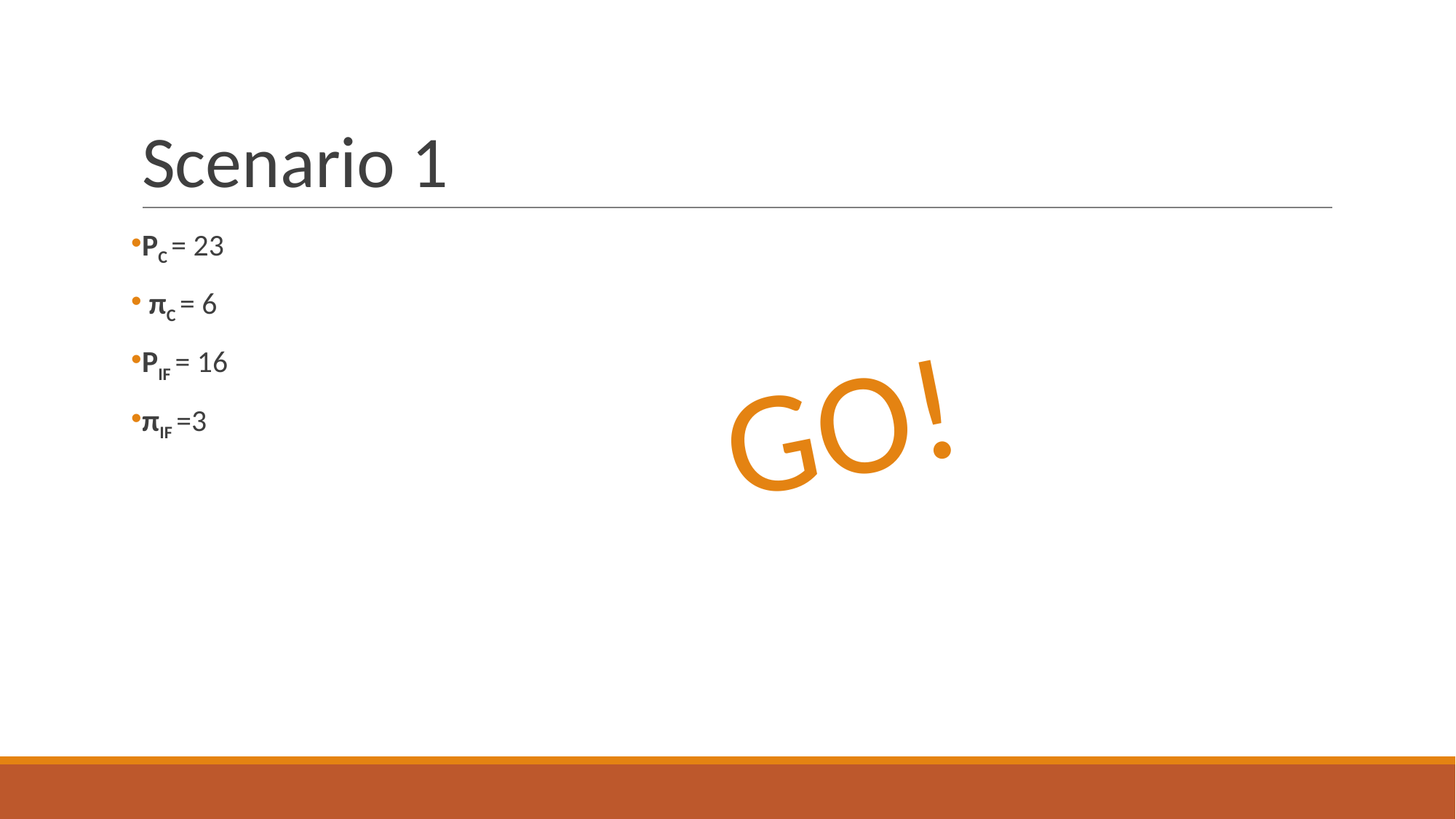

# Scenario 1
PC = 23
 πC = 6
PIF = 16
πIF =3
GO!

## Slide 9
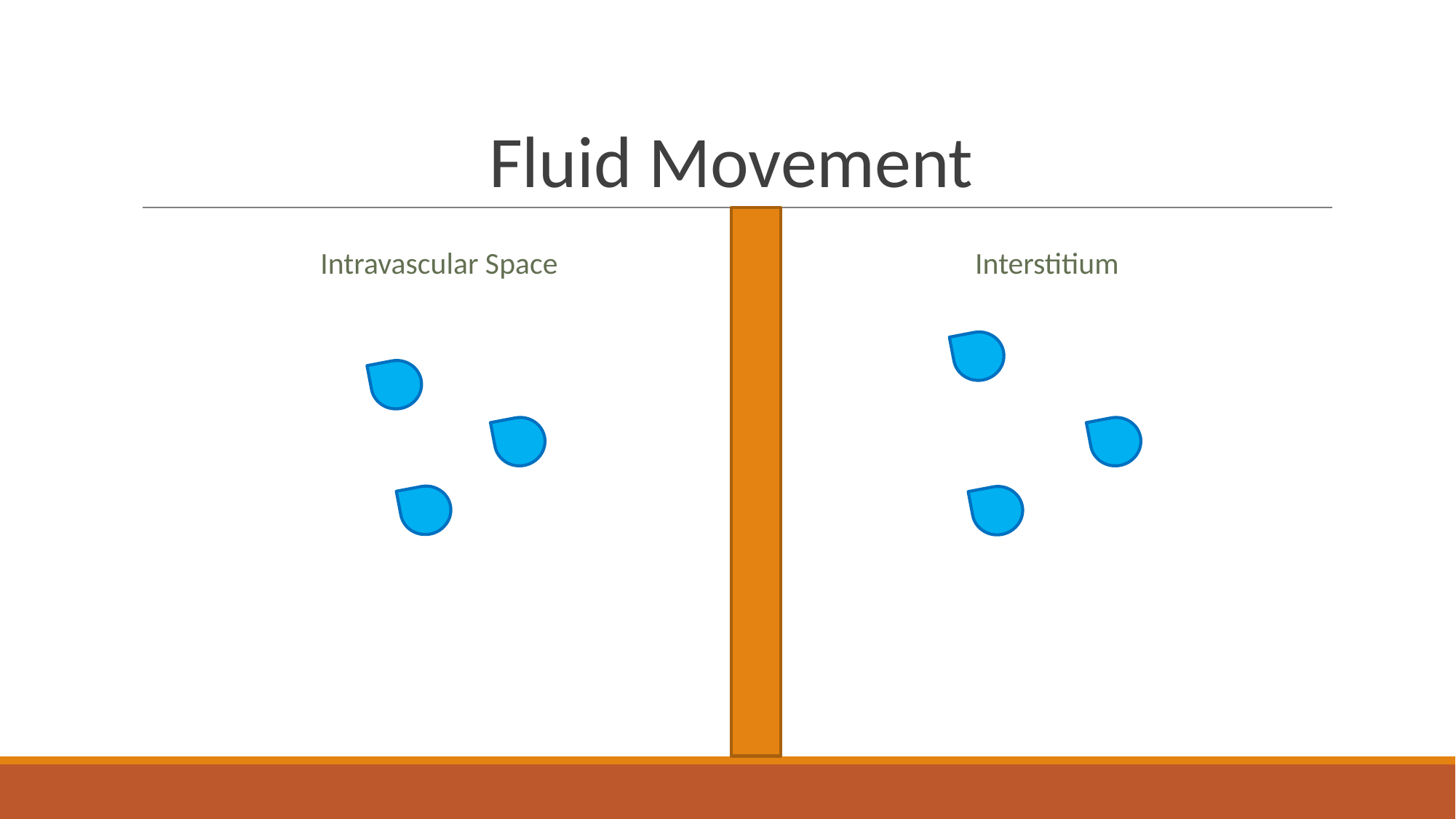

# Fluid Movement
Intravascular Space
Interstitium

## Slide 10
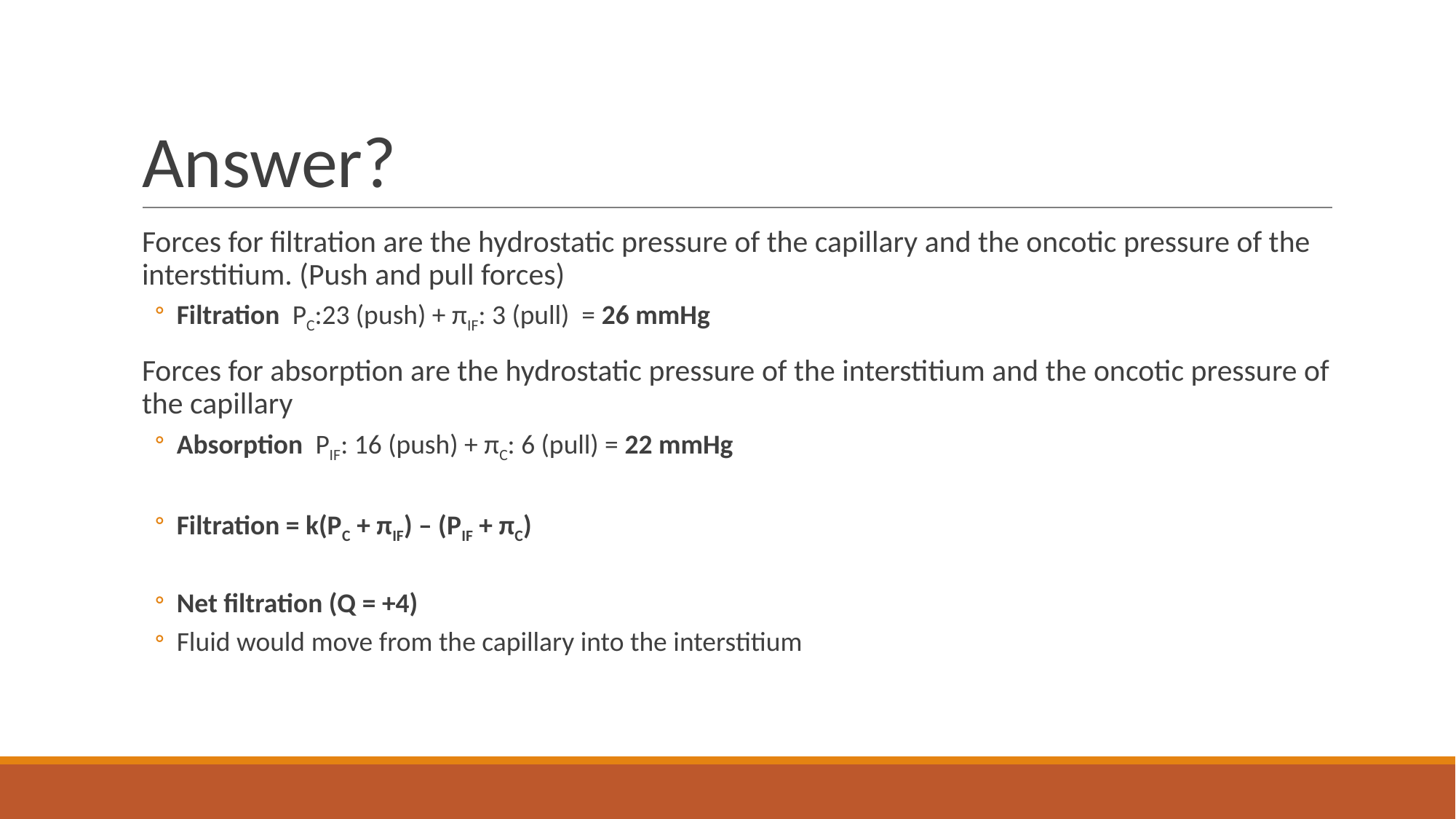

# Answer?
Forces for filtration are the hydrostatic pressure of the capillary and the oncotic pressure of the interstitium. (Push and pull forces)
Filtration PC:23 (push) + πIF: 3 (pull) = 26 mmHg
Forces for absorption are the hydrostatic pressure of the interstitium and the oncotic pressure of the capillary
Absorption PIF: 16 (push) + πC: 6 (pull) = 22 mmHg
Filtration = k(PC + πIF) – (PIF + πC)
Net filtration (Q = +4)
Fluid would move from the capillary into the interstitium

## Slide 11
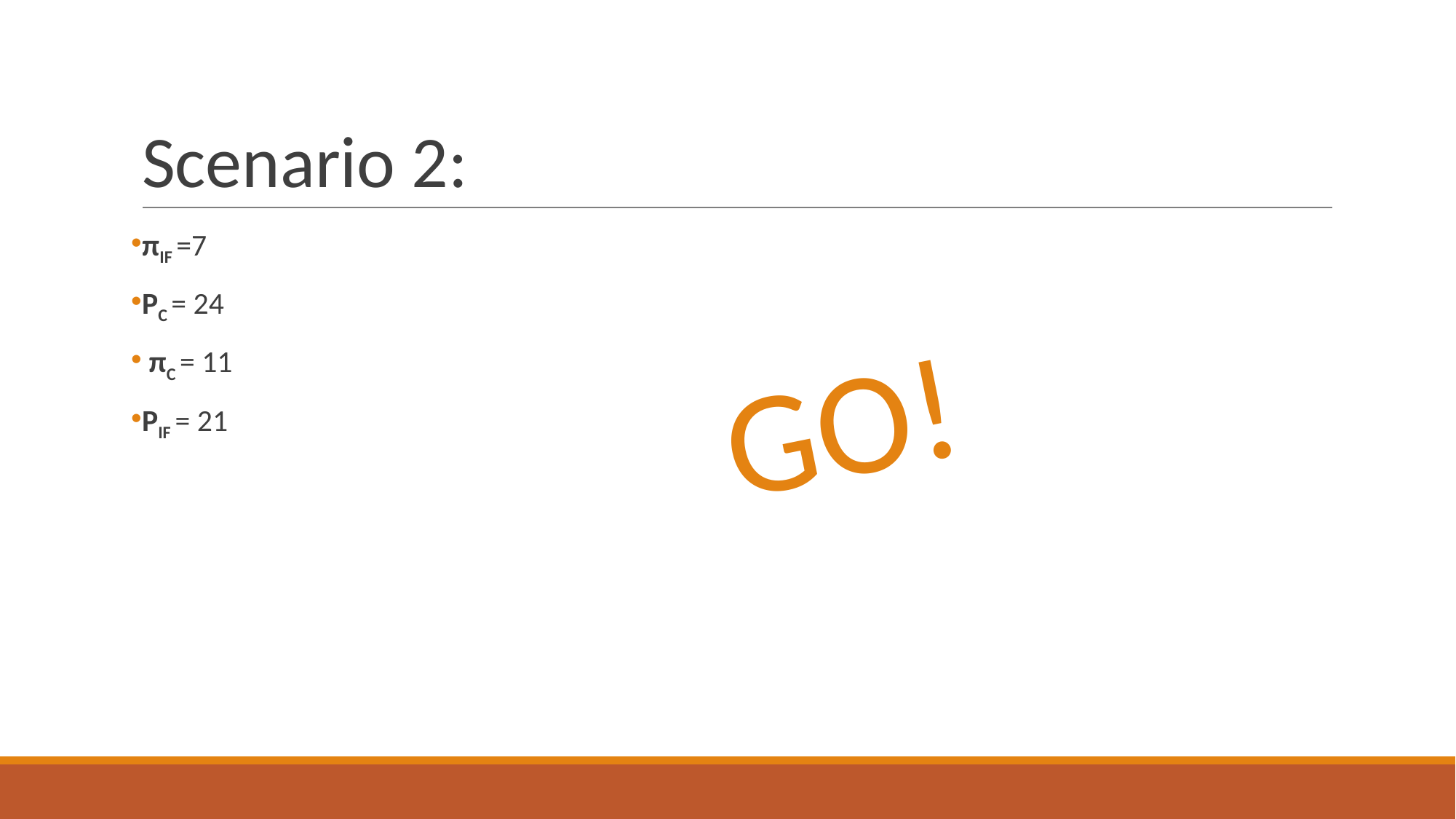

# Scenario 2:
πIF =7
PC = 24
 πC = 11
PIF = 21
GO!

## Slide 12
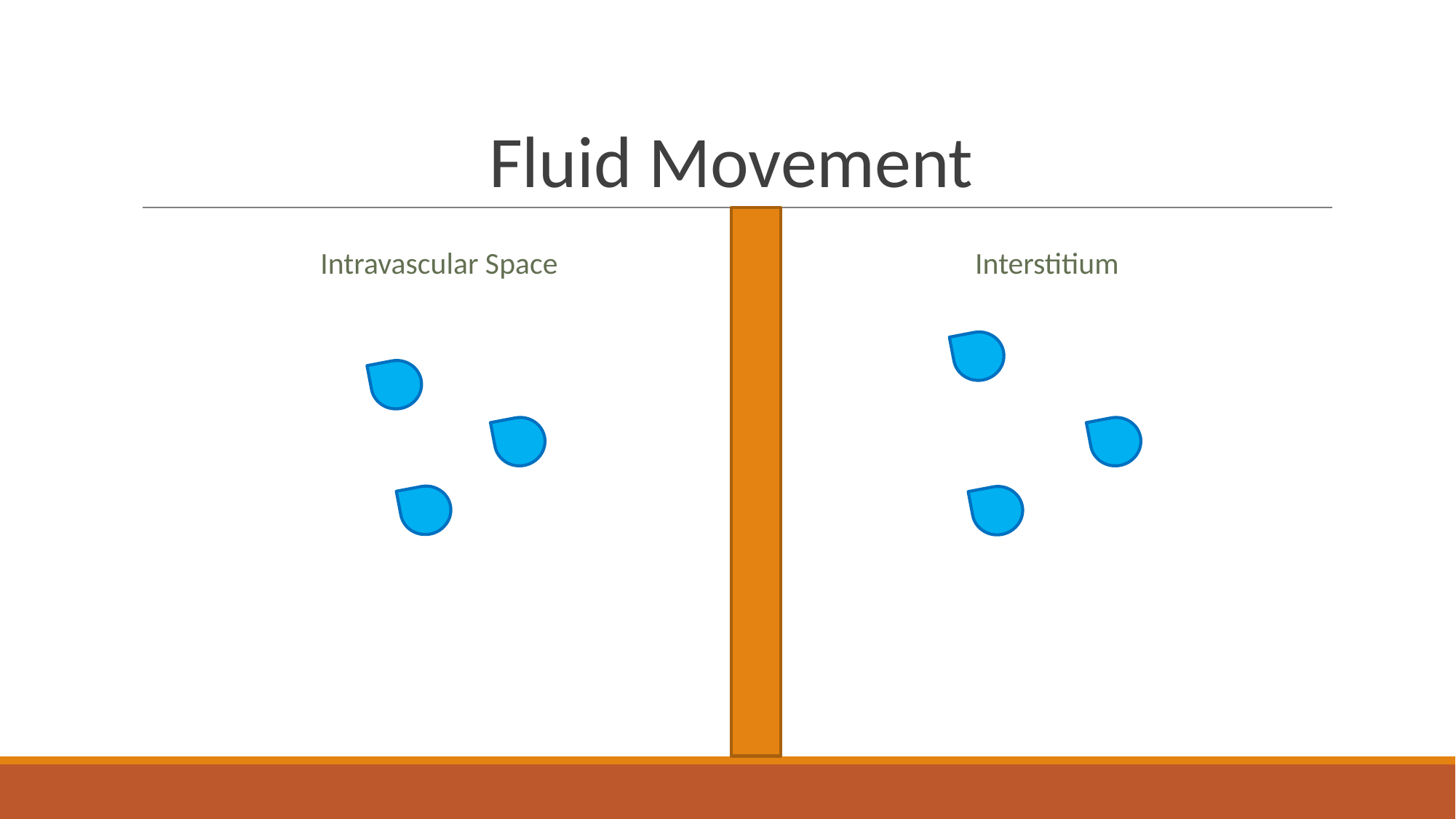

# Fluid Movement
Intravascular Space
Interstitium

## Slide 13
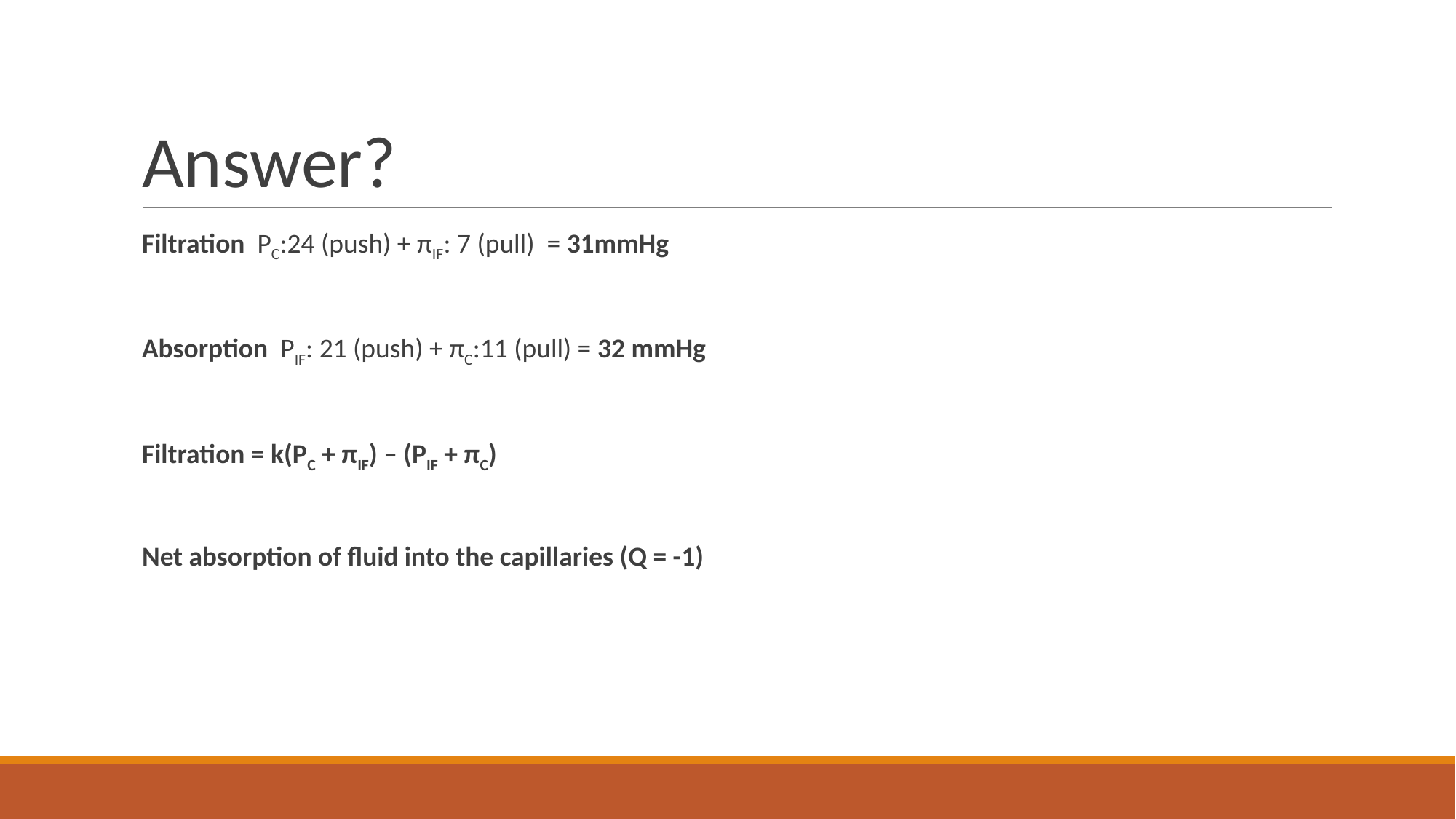

# Answer?
Filtration PC:24 (push) + πIF: 7 (pull) = 31mmHg
Absorption PIF: 21 (push) + πC:11 (pull) = 32 mmHg
Filtration = k(PC + πIF) – (PIF + πC)
Net absorption of fluid into the capillaries (Q = -1)

## Slide 14
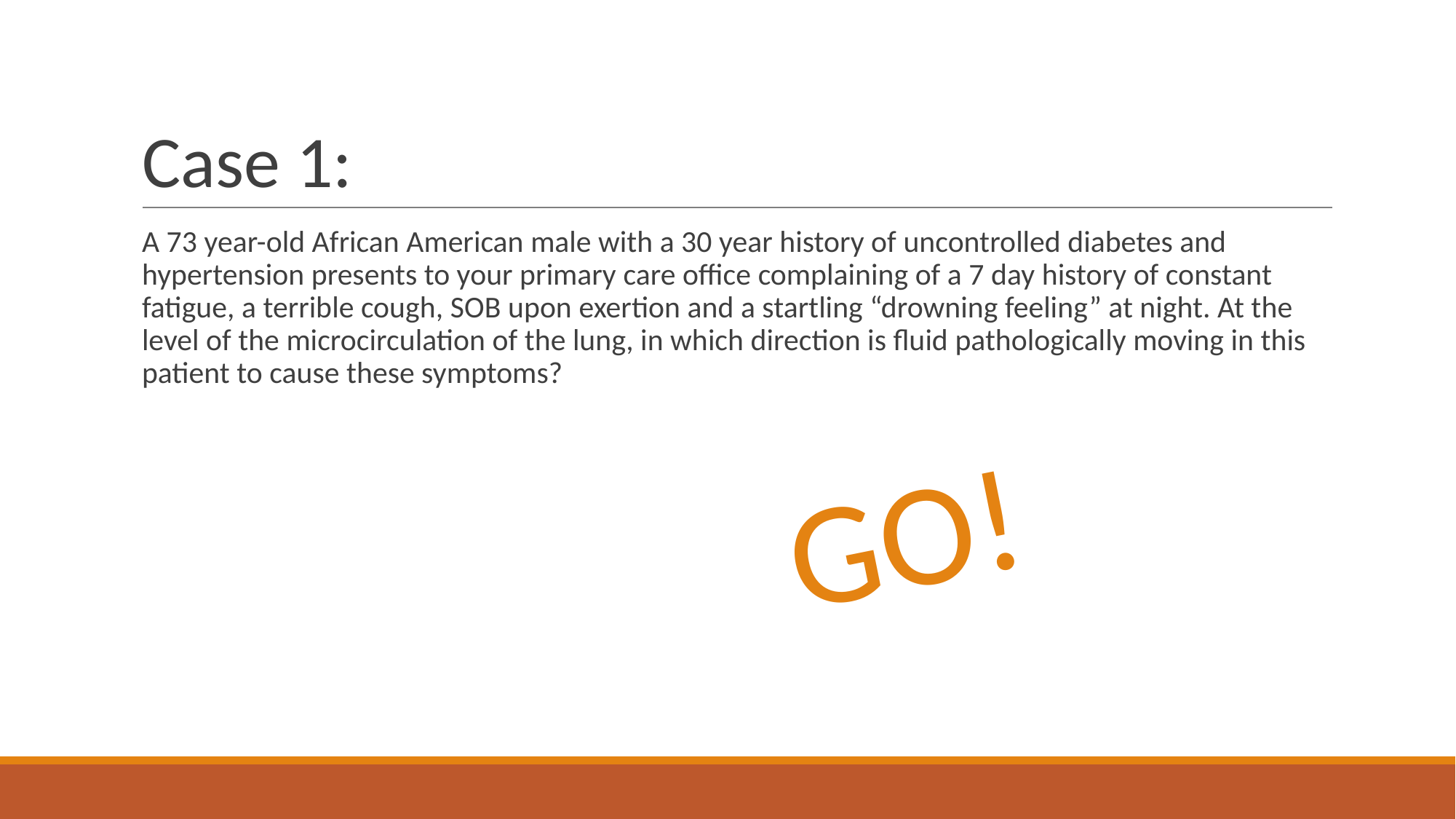

# Case 1:
A 73 year-old African American male with a 30 year history of uncontrolled diabetes and hypertension presents to your primary care office complaining of a 7 day history of constant fatigue, a terrible cough, SOB upon exertion and a startling “drowning feeling” at night. At the level of the microcirculation of the lung, in which direction is fluid pathologically moving in this patient to cause these symptoms?
GO!

## Slide 15
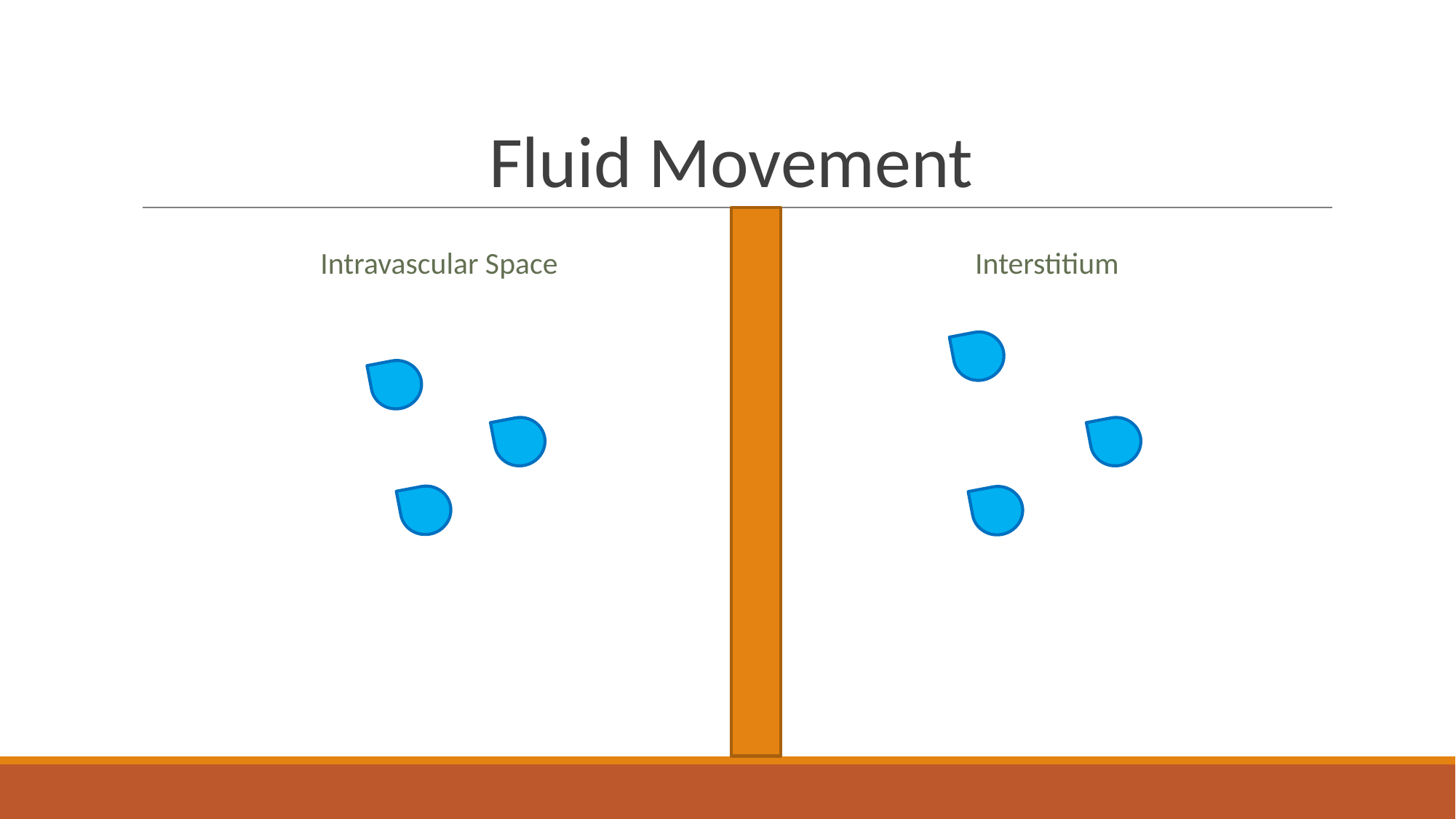

# Fluid Movement
Intravascular Space
Interstitium

## Slide 16
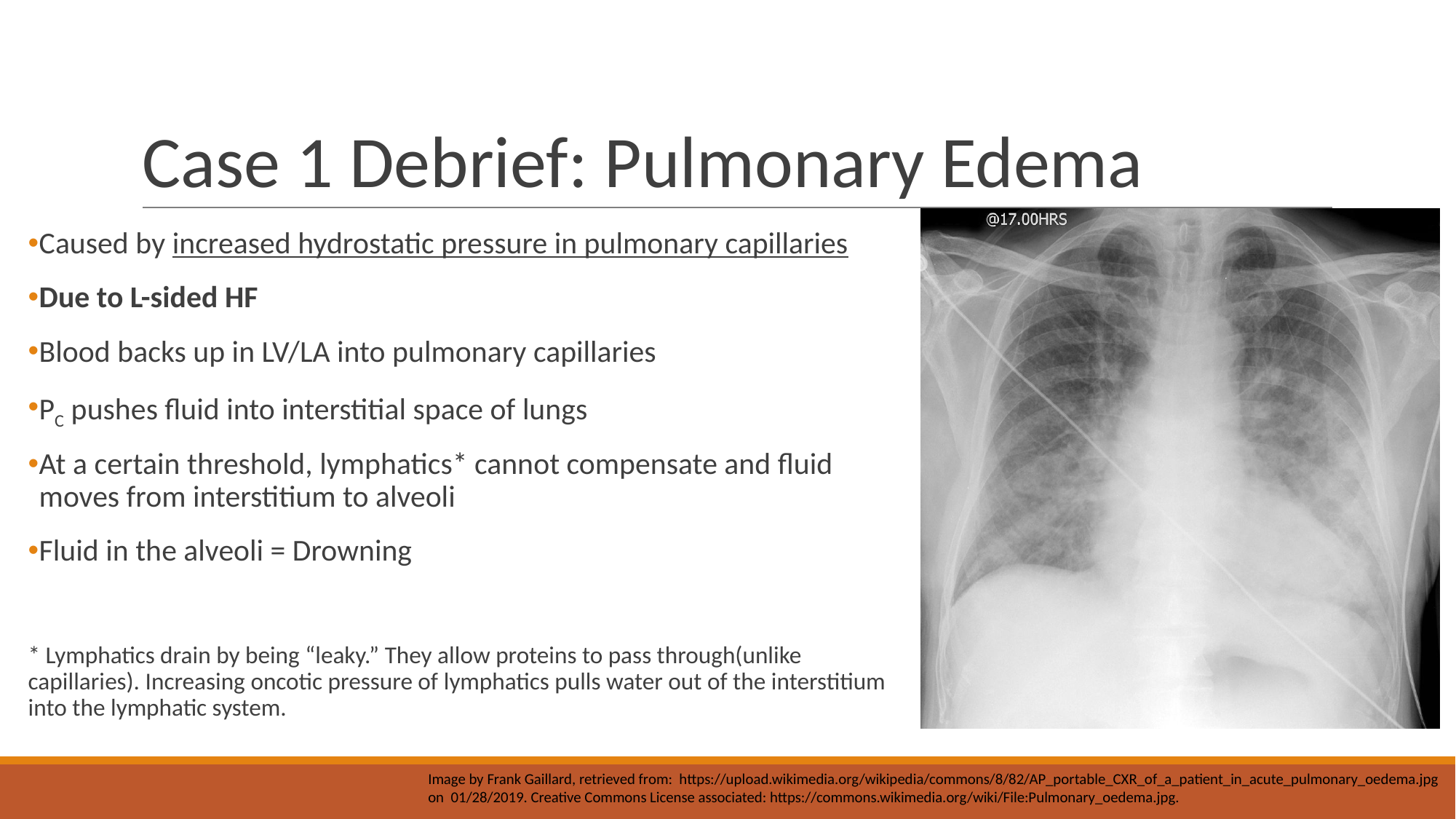

# Case 1 Debrief: Pulmonary Edema
Caused by increased hydrostatic pressure in pulmonary capillaries
Due to L-sided HF
Blood backs up in LV/LA into pulmonary capillaries
PC pushes fluid into interstitial space of lungs
At a certain threshold, lymphatics* cannot compensate and fluid moves from interstitium to alveoli
Fluid in the alveoli = Drowning
* Lymphatics drain by being “leaky.” They allow proteins to pass through(unlike capillaries). Increasing oncotic pressure of lymphatics pulls water out of the interstitium into the lymphatic system.
Image by Frank Gaillard, retrieved from: https://upload.wikimedia.org/wikipedia/commons/8/82/AP_portable_CXR_of_a_patient_in_acute_pulmonary_oedema.jpg
on 01/28/2019. Creative Commons License associated: https://commons.wikimedia.org/wiki/File:Pulmonary_oedema.jpg.

## Slide 17
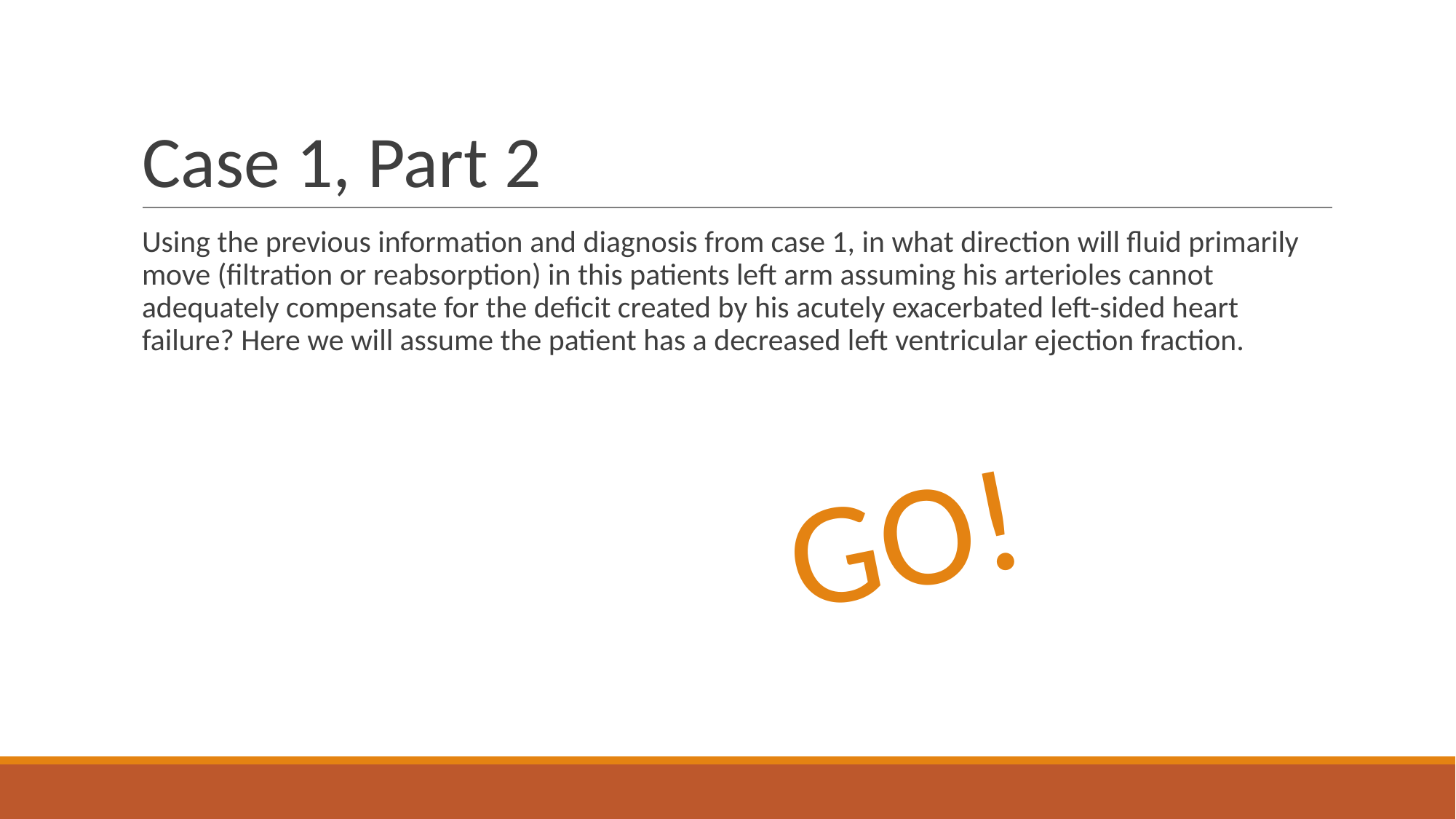

# Case 1, Part 2
Using the previous information and diagnosis from case 1, in what direction will fluid primarily move (filtration or reabsorption) in this patients left arm assuming his arterioles cannot adequately compensate for the deficit created by his acutely exacerbated left-sided heart failure? Here we will assume the patient has a decreased left ventricular ejection fraction.
GO!

## Slide 18
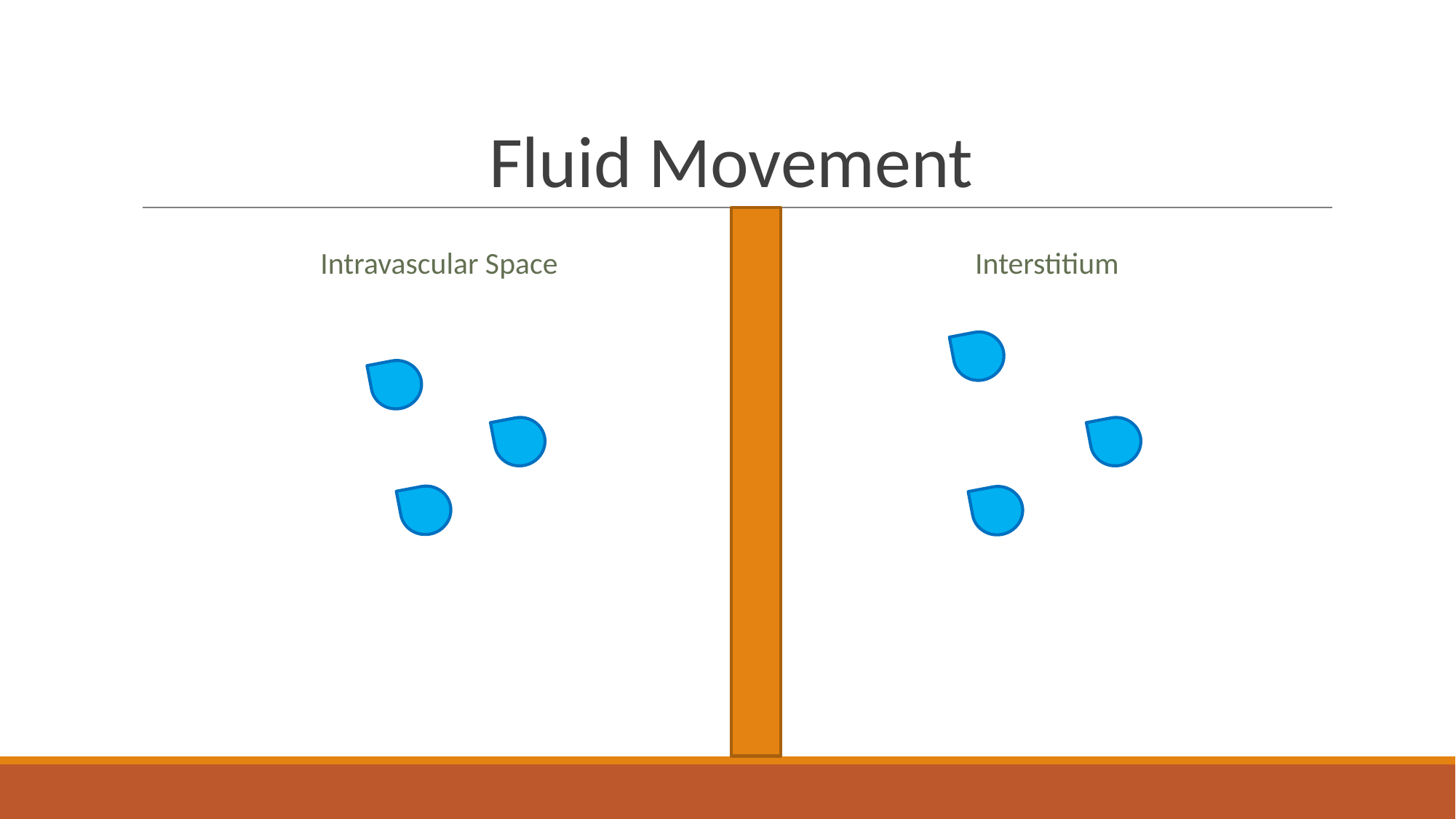

# Fluid Movement
Intravascular Space
Interstitium

## Slide 19
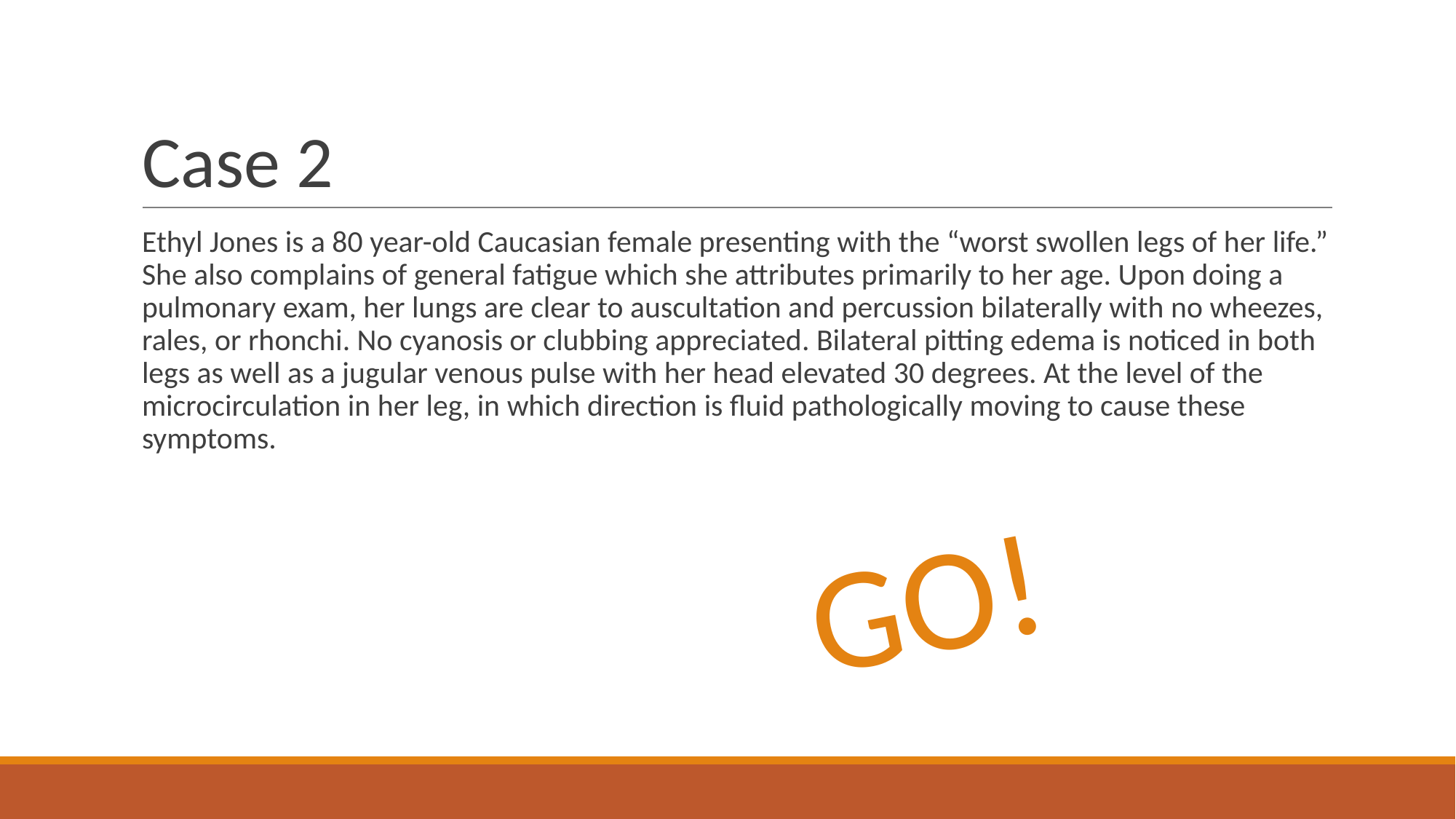

# Case 2
Ethyl Jones is a 80 year-old Caucasian female presenting with the “worst swollen legs of her life.” She also complains of general fatigue which she attributes primarily to her age. Upon doing a pulmonary exam, her lungs are clear to auscultation and percussion bilaterally with no wheezes, rales, or rhonchi. No cyanosis or clubbing appreciated. Bilateral pitting edema is noticed in both legs as well as a jugular venous pulse with her head elevated 30 degrees. At the level of the microcirculation in her leg, in which direction is fluid pathologically moving to cause these symptoms.
GO!

## Slide 20
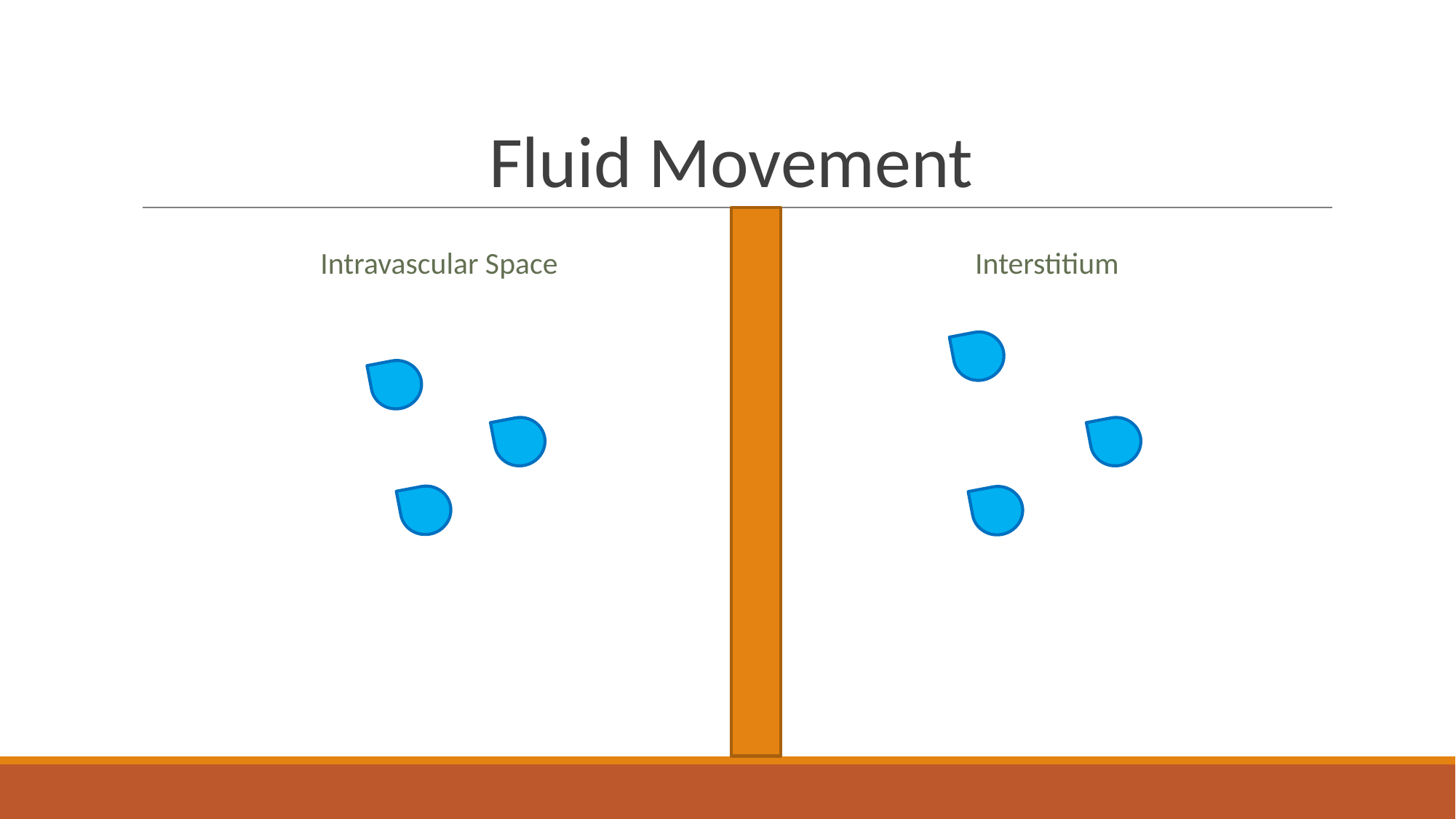

# Fluid Movement
Intravascular Space
Interstitium

## Slide 21
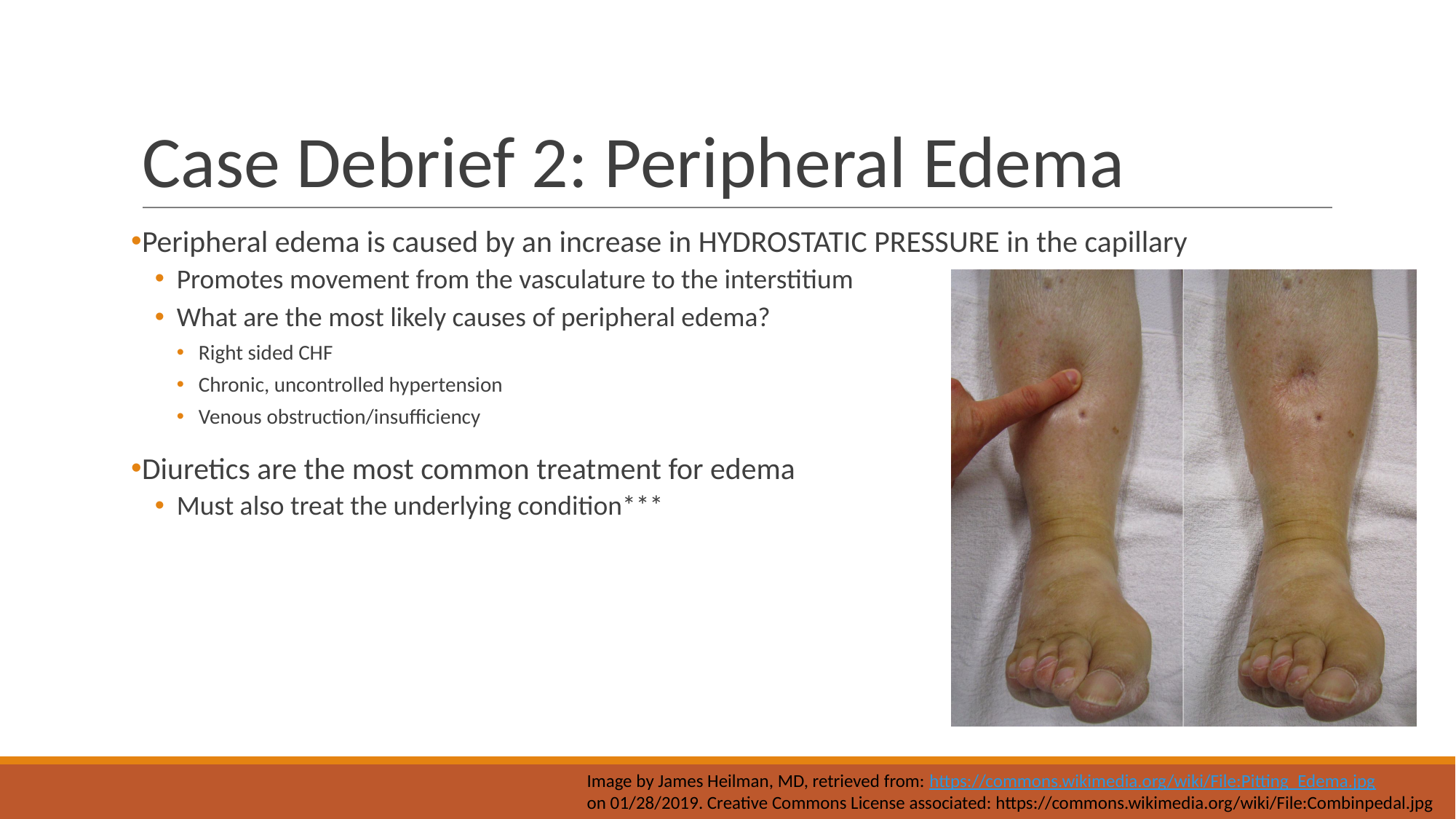

# Case Debrief 2: Peripheral Edema
Peripheral edema is caused by an increase in HYDROSTATIC PRESSURE in the capillary
Promotes movement from the vasculature to the interstitium
What are the most likely causes of peripheral edema?
Right sided CHF
Chronic, uncontrolled hypertension
Venous obstruction/insufficiency
Diuretics are the most common treatment for edema
Must also treat the underlying condition***
Image by James Heilman, MD, retrieved from: https://commons.wikimedia.org/wiki/File:Pitting_Edema.jpg
on 01/28/2019. Creative Commons License associated: https://commons.wikimedia.org/wiki/File:Combinpedal.jpg

## Slide 22
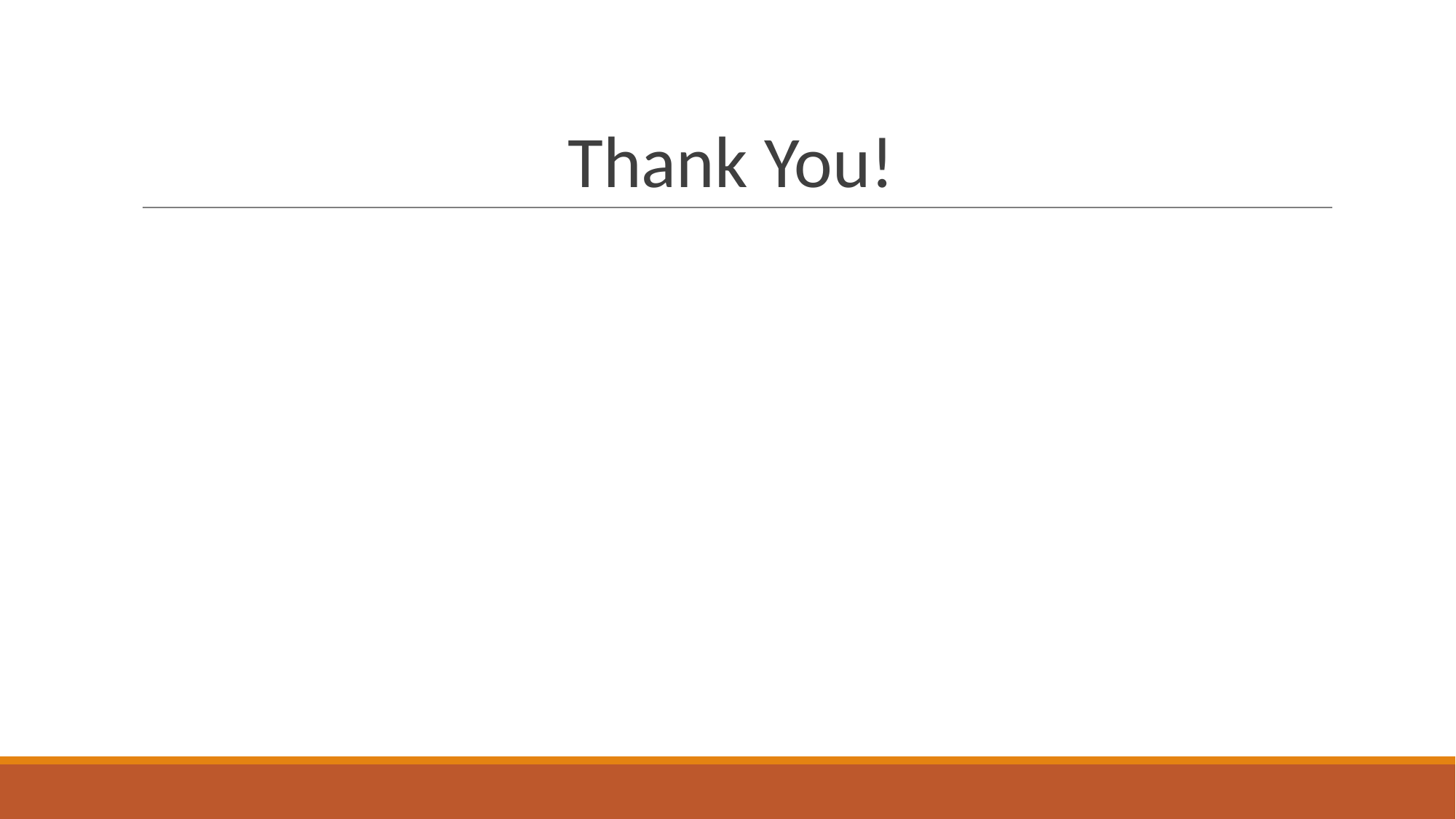

# Thank You!
