# Supplementary material for: Using Dramatization to Teach Starling Forces in the Microcirculation to First-Year Medical Students
Source: MedEdPORTAL. 2019 Oct 18;15:10842. doi: 10.15766/mep_2374-8265.10842 (PMC6944257; doi:10.15766/mep_2374-8265.10842)
Supplement: Supplementary file 1 — A. Starling Forces Workshop Lecture.pptx B. Preactivity Quiz.docx C. Postactivity Quiz.docx D. Preactivity Quiz Answers.docx E. Postactivity Quiz Answers.docx [file mep-15-10842-s001.zip › B. Preactivity Quiz.docx]

To compare performance before and after the actvity, please provide the month and date of your birthday in the upper right hand corner.. For example, October 5th = 1005.

Starling Forces Pre-Activity Evaluation

We want to invite you to participate in the ‘dramatization of Starling forces’ activity. The data collect from the surveys passed before and after the activity might be used for **research**. Please put the survey complete (or not) in the envelop provided. The activity is **voluntary** and **anonymous**. Your participation will not affect your grades.

1. Which of the Starling forces has the most pronounced effect upon fluid movement?

A. P_C_ (Capillary hydrostatic pressure)

B. π_C_  (Capillary oncotic pressure)

C. P_IF_ (Hydrostatic pressure of the interstitial fluid)

D. π_IF_ (Oncotic pressure of the interstitial fluid)

2. Which of the Starling forces favor the net filtration of fluid into the interstitium (P_C_, π_C_, P_IF,_ π_IF_) ?

1. Which of the Starling forces favor net absorption of fluid into the capillary?

1. A generally healthy 27 year-old female walks into your primary care office for a routine annual check up. The following values were obtained for the forces governing fluid movement:

P_C_ = 30, P_IF_ = 4, π_C_ =23, π_IF_ = 3

Calculate the value of Q (fluid flow) **and** state whether there will be net filtration or reabsorption at the level of the capillary at this moment in time.

1. At the level of the microcirculation, fluid pressure is maintained primarily by which type of blood vessel? And, in response to a systemic decrease in blood volume, these types of vessels will undergo (vasoconstriction or vasodilation) in an attempt to maintain a constant mean arterial pressure.

Vessel: _______________

Circle one: Vasoconstriction or Vasodilation

1. (T / F) The lymphatic system is responsible for the removal of excess fluid AND small proteins from the interstitium.
2. A 65 year-old caucasian male presents to the local Emergency Department complaining of sustained dyspnea over the past several hours. While in the ED, on several occasions, the patient struggled to say, ‘I feel like I’m drowning.’ On exam, no peripheral edema or JVD was noted.

What is the most likely cause for this man’s symptoms?

1. L-sided heart failure
2. R-sided heart failure
3. L and R-sided heart failure
4. An electrolyte imbalance
5. In the above case (number 7), assuming decreased renal perfusion with no accompaning renal damage, the kidneys would most likely compensate by
6. Increasing P_C_ via increased renin production
7. Decreasing P_C_ via decreased renin production
8. Decreasing P_C_ via increased renin production
9. Increasing P_C_ via decreased renin production
10. In the above case (number 7), what is the most likely mechanism resulting in the aforementioned symptoms?
11. Increased oncotic pressure of the interstitium, resulting in the net movement of fluid into the lungs
12. Decreased capillary hydrostatic pressure, resulting in the net movement of fluid into the capillary
13. Decreased capillary oncotic pressure, resulting in the net movement of fluid into the lungs
14. Increased capillary hydrostatic pressure, resulting in the net movement of fluid into the lungs
15. Decreased hydrostatic pressure of the interstitial fluid, resulting in the net movement of fluid into the lungs
16. A generally healthy 27 year-old female in her 32 week of gestation walks into your primary care office for her annual check up. The following values were obtained for the forces governing fluid movement in the peripheral vasculature:

P_C_ = 85, P_IF_ = 3, π_C_ =30, π_IF_ = 8

Given the above values, what is this woman’s most likely chief complaint?

1. Dizziness
2. Difficulty breathing
3. Leg pain
4. Stomach cramps

11. Which of the Starling forces do diuretics work to preferentially decrease?
 A. P_C_

B. π_C_

C. P_IF_

D. π_IF_
